# Profiling Microglia in a Mouse Model of Machado–Joseph Disease

**DOI:** 10.3390/biomedicines10020237

**Published:** 2022-01-23

**Authors:** Ana Bela Campos, Sara Duarte-Silva, Bruno Fernandes, Sofia Pereira das Neves, Fernanda Marques, Andreia Teixeira-Castro, Andreia Neves-Carvalho, Daniela Monteiro-Fernandes, Camila Cabral Portugal, Renato Socodato, Teresa Summavielle, António Francisco Ambrósio, João Bettencourt Relvas, Patrícia Maciel

**Affiliations:** 1 Life and Health Sciences Research Institute (ICVS), School of Medicine, University of Minho, 4710-057 Braga, Portugal; ana.bela.campos.88@gmail.com (A.B.C.); sarasilva@med.uminho.pt (S.D.-S.); pereiradasneves.sofia@mayo.edu (S.P.d.N.); fmarques@med.uminho.pt (F.M.); accastro@med.uminho.pt (A.T.-C.); andreiacarvalho@med.uminho.pt (A.N.-C.); id8942@alunos.uminho.pt (D.M.-F.); 2 ICVS/3B’s, PT Government Associate Laboratory, 4710-057 Braga, Portugal; 3 Department of Informatics, ALGORITMI Center, University of Minho, 4710-057 Braga, Portugal; bruno.fernandes@algoritmi.uminho.pt; 4 Glial Cell Biology Group, Instituto de Investigação e Inovação em Saúde (i3S), Instituto de Biologia Molecular e Celular (IBMC), University of Porto, 4200-135 Porto, Portugal; camila.portugal@ibmc.up.pt (C.C.P.); renato.socodato@ibmc.up.pt (R.S.); jrelvas@ibmc.up.pt (J.B.R.); 5 Addiction Biology Group, Instituto de Investigação e Inovação em Saúde (i3S), Instituto de Biologia Molecular e Celular (IBMC), University of Porto, 4200-135 Porto, Portugal; tsummavi@ibmc.up.pt; 6 ESS.PP, Escola Superior de Saúde do Politécnico do Porto, 4200-072 Porto, Portugal; 7 Coimbra Institute for Clinical and Biomedical Research (iCBR), Faculty of Medicine, Univiversity of Coimbra, 3004-504 Coimbra, Portugal; afambrosio@fmed.uc.pt; 8 Center for Innovative Biomedicine and Biotechnology (CIBB), Univiversity of Coimbra, 3004-504 Coimbra, Portugal; 9 Clinical Academic Center of Coimbra (CACC), 3004-561 Coimbra, Portugal; 10 Faculty of Medicine, University of Porto, 4200-319 Porto, Portugal

**Keywords:** microglia, Machado–Joseph disease, cell morphology, RNA-sequencing, machine learning

## Abstract

Microglia have been increasingly implicated in neurodegenerative diseases (NDs), and specific disease associated microglia (DAM) profiles have been defined for several of these NDs. Yet, the microglial profile in Machado–Joseph disease (MJD) remains unexplored. Here, we characterized the profile of microglia in the CMVMJD135 mouse model of MJD. This characterization was performed using primary microglial cultures and microglial cells obtained from disease-relevant brain regions of neonatal and adult CMVMJD135 mice, respectively. Machine learning models were implemented to identify potential clusters of microglia based on their morphological features, and an RNA-sequencing analysis was performed to identify molecular perturbations and potential therapeutic targets. Our findings reveal morphological alterations that point to an increased activation state of microglia in CMVMJD135 mice and a disease-specific transcriptional profile of MJD microglia, encompassing a total of 101 differentially expressed genes, with enrichment in molecular pathways related to oxidative stress, immune response, cell proliferation, cell death, and lipid metabolism. Overall, these results allowed us to define the cellular and molecular profile of MJD-associated microglia and to identify genes and pathways that might represent potential therapeutic targets for this disorder.

## 1. Introduction

Microglia, the primary immune cells of the central nervous system (CNS), play multiple roles in neurodevelopment, synaptic plasticity, homeostasis, injury responses [1,2], and neurodegenerative diseases (NDs) [3]. Microglia can polarize into different activation states depending on the gamut of environmental cues they are exposed to [4,5]. However, defining microglial transcriptomic signatures in different states has revealed that their activation profile is quite heterogeneous and, to a large extent, context dependent [6].

The morphological characterization of microglia is of the utmost importance, as it is widely used to define their activation status and changes substantially under brain disease and pathology. Ramified microglia can undergo morphological transformations into an “activated state” (characterized by larger cell bodies with shorter and thicker processes) [6,7] or a “reactive state” (characterized by smaller, spherical cells that can also exhibit rod-shaped or amoeboid-like morphologies) [6,7,8,9].

Age-related neurodegenerative diseases are associated with chronic neuroinflammation, and microglia-mediated inflammation is a significant contributor to disease pathogenesis [10,11,12,13,14]. Aging causes microglia to adopt an aberrant phenotype, sometimes referred to as dystrophic or senescent, usually associated with a decreased ability to provide a normal response to injury [11]. Cellular senescence is typically characterized by an arrested growth due to elevated DNA damage and oxidative stress that increases the amounts of senescence indicators, including the cell cycle regulators P16^Ink4a^ (also known as *Cdkn2a*), P19^Arf^ (also known as *Cdkn2a*) and P21^Cip1/Waf1^ (also known as *Cdkn1a*), and pro-inflammatory cytokines, such as Pai1 (also known as *Serpine1*), Il-6, Il-8, Il-1 alpha, and Il-1 beta [15]. Reduced phagocytic capacity [11,16], impaired protein homeostasis (proteostasis) [17], and dystrophic morphology, characterized by de-ramification and shortening of the processes [18], are also consistent age-related changes in microglia. These changes may contribute to an increased susceptibility to neuronal dysfunction and demise during aging, through increased production of inflammatory mediators and impairment of microglia neuroprotective functions [4,14].

Little is known about the profile of microglia and their involvement in Machado–Joseph disease (MJD), a neurodegenerative disorder caused by an abnormal expansion of a CAG triplet that encodes the amino acid glutamine in the ataxin-3 protein [19]. The CAG repeat size in the *ATXN3* gene translates into a polyglutamine tract of 61 to 87 glutamines that renders ataxin-3 prone to self-assembly and thus to the formation of aggregates that are toxic to neurons [19,20]. While ataxin-3 misfolding and the consequent disruption of cells’ proteostasis are considered central to MJD pathogenesis [20], transcriptional dysregulation, oxidative stress, and DNA damage also contribute to disease progression [20]. Neuropathological analyses of MJD patients’ brains reveal significant neuronal loss in the deep cerebellar nuclei (DCN) within the cerebellum, pontine nuclei (PN) within the brainstem, and in spinocerebellar tracts. The motor symptoms appear gradually and progress over time, pointing to an age-dependent decline in the cells’ ability to remove misfolded proteins [21,22]. Although microgliosis has been observed both in MJD patients’ post-mortem brains [23,24,25] and a mouse model of MJD [26], further studies are required to fully understand the basis of microglial activation in MJD [25]. Because most brain cells express *ATXN3*, microglial dysfunction may contribute to the disease process due to the effects of mutant *ATXN3* in microglia or as a consequence of their interaction with neurons.

In this study, we used the CMVMJD135 mouse model [27] to characterize the profile of microglia in the context of MJD. Combining principal components analysis (PCA), machine learning models, and RNA sequencing, we characterized morphological clusters and mapped gene expression networks in MJD-derived microglia, providing relevant novel insights into how coordinated microglia morphology and gene regulatory programs might contribute to MJD pathogenesis.

## 2. Materials and Methods

### 2.1. Animal Maintenance

CMVMJD135 and wild-type (WT) littermates’ mice on a C57BL/6J background were used. The CMVMJD135 mouse expresses an expanded version of the human MJD1-1 cDNA (the 3 UIMs-containing a variant of ataxin-3) under the regulation of the CMV promoter (ubiquitous expression) at near-endogenous levels and manifests MJD-like motor symptoms that appear gradually and progress over time [27,28]. All animals (specific pathogen-free health status) were maintained under standard laboratory conditions: an artificial 12 h light/dark cycle (lights on from 8:00 to 20:00 h), with an ambient temperature of 21 ± 1 °C and a relative humidity of 50–60%. All animal procedures were conducted following the European Union Directive 2010/63/EU. Health monitoring was performed according to the Federation of European Laboratory Animal Science Associations (FELASA) guidelines. The specified pathogen-free health status was confirmed by sentinel mice maintained in the same animal housing room. Except for the primary culture of microglial cells that used 3-to-4-day-old (P3-P4) WT and CMVMJD135 mice, all the remaining experiments were performed using animals of 34–50 weeks of age, corresponding to an advanced disease stage.

### 2.2. Evaluation of Microglia Phagocytic Ability in Culture

After the characterization of a microglia-enriched culture (detailed protocol in Supplementary Materials and Methods), their phagocytic activity and morphology were assessed, as described in [11], in two conditions: basal and exposed to lipopolysaccharide (LPS, E. coli O111:B4, Sigma-Aldrich, St. Louis, MO, USA), and at two different time points, 4 and 16 days in vitro (DIV), as presented in Table S1 in Supplementary Materials and Methods.

To evaluate the phagocytic activity of the primary microglial cultures, the cells, collected at two different time points (4 and 16 DIV), were incubated with 0.0025% (*w*/*w*) of 1 μm green fluorescent latex beads (Sigma-Aldrich, St. Louis, MO, USA). For immunofluorescence detection, the cells were fixed for 15 min with freshly prepared 4% paraformaldehyde (PFA) in phosphate saline buffer (PBS), permeabilized with 0.1% Triton X-100 for 20 min, and then blocked with PBS containing 2% bovine serum albumin (BSA) for 1 h. After this, the microglial cells were incubated with an anti-ionized calcium binding adaptor molecule 1 (Iba-1) antibody (Table S2 in Supplementary Materials and Methods) overnight at 4 °C, followed by secondary antibody incubation (anti-rabbit Alexa Fluor 594, Table S2 in Supplementary Materials and Methods) for 2 h at room temperature (RT). Cell nuclei were stained with 4’,6-diamidin-2-phenylindol (DAPI, Invitrogen, Waltham, MA, USA) for 10 min at RT. Random fluorescence images (7 to 22) were acquired per coverslip, animal, condition, and experimental group (Table S2 in Supplementary Materials and Methods), using an Olympus Widefield Inverted Microscope IX81 (Olympus Corporation, Tokyo, Japan) (resolution of 1024 × 1024 px and an original magnification of 20×).

To evaluate the phagocytic capacity of the primary microglial cultures, the number of ingested beads per cell was counted using the Point Tool feature in ImageJ software (v1.53c). Results are presented as phagocytic efficiency, considering the total number of microglial cells, to obtain the average amount of ingested beads per cell, considering the proportion of cells phagocytosing 1, 2, 3, 4, 5, and more than 5 beads, obtained by the formula described in [29].

### 2.3. Evaluation of Microglial Morphology in Culture

For the morphological analysis, cells were fixed with 4% PFA in PBS, and a standard immunolabeling technique was performed using a primary antibody against Iba-1 to evaluate the microglia phagocytic ability. To identify the cells, microglial nuclei were stained with DAPI. Using ImageJ software, cells were outlined with the Freehand Selection tool. Then the particle measurement feature was used to automatically measure the 2D area, perimeter, and the Feret’s diameter of at least 3 single microglial cells per image [11]. The fluorescence images used to evaluate phagocytic capacity were also used to characterize the microglia morphology, quantitatively. The transformation index, which categorizes the microglia ramification status, was also assessed as described previously [30].

### 2.4. Quantitative Reverse-Transcription PCR (qRT-PCR)

To evaluate the mRNA expression levels of human *ATXN3*, RNA was extracted from CMVMJD135 and WT neonatal mice-derived microglial. To evaluate senescence marker levels, RNA was extracted from CMVMJD135 and WT tissues previously frozen (brainstem, cerebellum, and spinal cord). TRIZOL (Invitrogen, Waltham, MA, USA) was used in both cases, following the manufacturer’s instructions. Samples were treated with DNase I (ThermoFisher Scientific, Waltham, MA, USA); RNA concentration was quantified using the NanoDrop™ Spectrophotometer (ThermoFisher Scientific, Waltham, MA, USA), and RNA quality was tested through electrophoresis. Afterwards, 1 μg first-strand complementary DNA (cDNA) was synthesized using the iScript™ cDNA synthesis kit (Bio-Rad, Hercules, CA, USA). The quantitative polymerase chain reaction (PCR) was then carried out using the 5× HOT FIREPol^®^ EvaGreen^®^ qPCR Mix Plus (ROX) (Solis BioDyne, Tartu, Estonia) with 1 μL of cDNA. Specific primers for different messenger RNAs were obtained either from the literature or those previously designed by us, using Primer-BLAST. The used primers are listed in Table S3 of Supplementary Materials and Methods. The housekeeping genes, Beta-2-microglobulin (*B2m*) or endogenous mouse *Atxn3*, were used as an internal standard to normalize the expression of selected transcripts. PCR reaction was run in Applied Biosystems™ 7500 Real-Time PCR System, and raw data were extracted using 7500 Real-Time PCR software v2.3 (Applied Biosystems by ThermoFisher, Waltham, MA, USA). All melting curves exhibited a single sharp peak at the expected temperature. Statistical analysis was conducted using 2^−ΔCT^ values, and plots were reported in fold change (2^−ΔΔCT^) or reported as fold change normalized to the mean of the relative expression of the control group.

### 2.5. Flow Cytometry Analysis

Microglia were collected from the affected brain regions as a whole (cerebellum, brainstem, and spinal cord) of WT and CMVMJD135 littermates, using density gradient separation. The following markers were used to characterize these cells in the samples: CD45-PE, CD11b-PE/Cy7, and CD11b-Alexa Fluor 647.

For the intracellular analysis of the P19^Arf^ and P21^Cip1/Waf1^ senescence markers, microglia were fixed, permeabilized, and incubated with anti-rat P19^Arf^ and anti-rabbit P21^Cip1/Waf1^ antibodies (Table S2 in Supplementary Materials and Methods). Briefly, mice were deeply anesthetized with a mixture of ketamine hydrochloride (150 mg/kg) and medetomidine (0.3 mg/kg) and perfused with ice-cold PBS. The tissues were quickly dissected and mechanically homogenized. The cell suspension was passed through a 100 μm cell strainer and centrifuged over a discontinuous 70/30% Percoll (GE Healthcare, Chicago, IL, USA) gradient. Single-cell suspensions (5 × 10^5^ cells) were seeded in a U-shape bottom 96-well plate and incubated with CD45-PE, CD11b-Alexa Fluor 647, or CD11b-PE/Cy7 for 30 min at 4 °C in the dark. After antibody washing, cells were fixed in 2% PFA for 30 min and permeabilized with a permeabilization buffer (Life Technologies, Carlsbad, CA, USA). Intracellular staining mix using the anti-rat P19^Arf^ and anti-rabbit P21^Cip1/Waf1^ antibodies was prepared in a permeabilization buffer. Microglia were then incubated with this intracellular staining mix overnight, at 4 °C in the dark. After that, cells were incubated with Alexa Fluor 488 and 647 secondary antibodies for 1 h at RT in the dark.

For intracellular detection of Il-8, Il-6, Il-1 alpha, and Il-1 beta, cells were incubated with 10 μg/mL of brefeldin A (Sigma-Aldrich, St. Louis, MO, USA) over 3 h, in an RPMI medium supplemented with 10% FBS and 1% antibiotic–antimycotic solution, and maintained at 37 °C in a humidified atmosphere of 5% CO_2_. After staining for the expression of surface molecules, cells were fixed with 2% PFA and permeabilized with a permeabilization buffer. After permeabilization, the cells were stained with anti-rabbit Il-6 and anti-mouse Il-8, or anti-mouse Il-1 alpha and anti-rabbit Il-1 beta antibodies overnight, at 4 °C, followed by Alexa Fluor 488 and 647 secondary antibodies (Table S2 in Supplementary Materials and Methods) for 1 h at RT in the dark.

Data acquisition was performed in Fluorescence-Activated Cell Sorting (FACS) Canto II analyzer (BD Immunocytometry Systems, San Jose, CA, USA) and data were analyzed by FlowJo X10 software (FlowJo, Ashland, OR, USA).

### 2.6. Tissue Preparation and Immunofluorescence Staining

CMVMJD135 and WT littermates were deeply anesthetized and transcardially perfused with PBS followed by 4% PFA solution (PFA, 0.1 M, pH 7.4, in PBS). Brain tissue was removed and fixed in a 4% PFA for 48 h, followed by 30% sucrose solution for 1 week. Then, coronal, and sagittal sections of 40 μm thickness were sliced using a Leica Vibratome. Tissue slices were permeabilized with PBS-T 0.3% (0.3% Triton X-100; Sigma Aldrich, St. Louis, MO, USA) for 10 min. Antigen retrieval was then performed by immersing the slices in pre-heated citrate buffer (10 mM, pH 6.0; Sigma Aldrich, St. Louis, MO, USA) for 20 min using a thermoblock (D1200, LabNet, Cary, NC, USA) set at 80 °C. Once cooled, the slices were rinsed in PBS and then blocked with goat serum blocking buffer (10% normal goat serum (NGS), 0.3% Triton X-100, in PBS) at RT for 90 min. After that, the slices were incubated with the primary antibody rabbit anti-Iba-1 diluted in PBS-T 0.3%, 5% NGS, overnight at 4 °C. Then, the tissue slices were rinsed in PBS and incubated with a secondary antibody, Alexa Fluor 594 anti-rabbit (Table S2 in Supplementary Materials and Methods) diluted in PBS-T 0.3%, 5% NGS, for 90 min at RT, protected from light. The sections were mounted on microscope slides (Menzel Gläser Superfrost^©^Plus, ThermoFisher Scientific, Waltham, MA, USA) and covered with a coverslip (Menzel Gläser 24–60 mm, Wagner & Munz, Munchen, Germany) using an aqueous mounting medium (Fluoromount™ Sigma-Aldrich, St. Louis, MO, USA).

### 2.7. Image Acquisition for Microglial Density and Morphological Analysis

For the analysis of microglial density and morphology, four coronal brain sections per animal (n=4 per genotype) were imaged twice (in both hemispheres) for each region of interest (DCN and cervical spinal cord (CSC)) to yield 4–6 digital photomicrographs per section containing the region for analysis. For the PN, four sagittal brain sections per animal were used (n=3 animals for WT and n=4 animals for CMVMJD135), and 2 photomicrographs per section were taken. The Olympus Confocal FV1000 laser scanning microscope with a resolution of 1024 × 1024 px using a 40× objective (UPlanSApo, N.A. 0.90; dry; field size 624.39 × 624.39 μm; 0.31 μm/px) was used to obtain all Z-stacked images. The acquisition settings were the following: scanning speed = 4 μm/px; pinhole aperture = 110 μm; Iba-1, excitation = 559 nm, emission = 618 nm; in a 3-dimensional scenario (X, Y, and Z axes).

The ImageJ software was used on Z-stacked 3D volume images from sections of the affected brain regions (DCN, CSC, and PN). The total count of Iba-1-positive cells was obtained using the multi-point tool of ImageJ. Quantification was performed on images acquired with acquisition settings described as above, normalized first to the total image area and then for volume. For morphological analysis, a semi-automatic method adapted from [31] was used (detailed protocol in Supplementary Materials and Methods and Figure S1 in Supplementary Materials and Methods).

### 2.8. Morphological Data Acquisition and Pre-Processing

When performed manually over every cell, obtaining all morphological features is demanding and laborious. Hence, to expedite the process, the *MorphData* plugin was used [32]. This plugin automatizes the data extraction process of morphological features of single microglial cells, collecting, pre-processing, and organizing features associated with cell complexity and ramification (detailed protocol in Supplementary Materials and Methods and Figures S2 and S3 in Supplementary Materials and Methods).

Data were obtained from individual cells of the CSC (310 microglial cells from WT mice and 389 from CMVMJD135 mice), DCN (349 microglial cells from WT mice and 445 from CMVMJD135 mice), and PN (152 microglial cells from WT mice and 180 from CMVMJD135 mice). The total number of analyzed microglia was 1825.

### 2.9. Machine Learning Modeling

An open-source data science and machine learning modeling platform was used to further process the obtained data and identify potential microglia clustering, concerning their morphological features. KNIME is a data-flow-centric platform, enabling visual and interactive flows.

Using the KNIME platform, two flows were conceived. The first is responsible for loading the obtained data and applying functions to arrange identical data into groups. As explained in subsequent lines, these data are then used for statistical analysis. The second flow is responsible for applying a PCA to the obtained data. This flow is also used to apply an unsupervised machine learning model, the k-means, a clustering method that finds groups or clusters with similar characteristics within the entire dataset. This method partitions the data into *k* clusters, with each observation belonging to a single cluster, represented by its centroid. To find the ideal number of clusters, i.e., the ideal number for *k*, the flow applies the elbow method, experimenting and plotting the mean squared error (MSE) associated with each cluster, with *k* varying between 1 and 12. The ideal *k* is found by picking the “elbow” of the curve as a function that minimizes the error of *k*. This flow is also used to generate 3D plots.

Finally, gradient boosted trees were used to obtain estimates of parameter importance, i.e., a score that measures how valid each parameter is for the model. Gradient boosted trees are a supervised machine learning model used to convert weak learners, typically decision trees, into strong ones. Gradient boosted trees train the learners gradually, additively, and sequentially, performing a gradient descent procedure. The importance was estimated using gain as the importance type. A higher value for a parameter, when compared to another, implies it is more important for classifying the label [33]. In this case, the label was set as the parameter identifying WT and CMVMJD135 cells, a binary classification problem.

### 2.10. Brain Dissociation for Magnetic Activated Cell Sorting Isolation of Adult Microglia

#### 2.10.1. Cellular Suspension Preparation

Microglia were isolated from the brainstem and cerebellum of WT and CMVMJD135 mice as described in [34]. The isolation was performed by pooling these 2 brain areas from 3 animals for each experiment. Hence, n=5 implies the use of 15 WT animals and 15 CMVMJD135 ones. Mice were transcardially perfused under deep anesthesia with PBS. Then the brainstem and cerebellum were removed, dissected, and rinsed in cold Hanks’ Balanced Salt solution without calcium chloride or magnesium chloride (HBSS[-]CaCl_2_/[-]MgCl_2_; ThermoFisher Scientific). The regions of interest were cut into small pieces using a sterile scalpel, and the samples were centrifuged at 300×g for 2 min at 4 °C, and the supernatant was discarded carefully. According to the manufacturer’s instructions, enzymatic cell dissociation was performed using a neural tissue dissociation Kit (Miltenyi Biotec, Cologne, Germany). Briefly, the enzyme mix 1 (50 μL of enzyme P and 1950 μL of buffer x), previously vortexed and pre-heated at 37 °C for 15 min, was transferred to the tissue pieces (up to 400 mg of tissue per sample). Then we proceeded to incubate for another 15 min at 37 °C under slow rotation to allow the digestion of the tissue. The enzyme mix 2 (10 μL of enzyme A and 20 μL of buffer Y) was then added, and the tissue was dissociated mechanically using a 1 mL syringe and a 20 G needle. After that, the samples were resuspended with cold Hanks’ Balanced Salt solution with calcium chloride and magnesium chloride (HBSS[+]CaCl_2_/[+]MgCl_2_; ThermoFisher Scientific, Waltham, MA, USA) and filtered through a 70 μm cell strainer (Sigma-Aldrich, St. Louis, MO, USA) to remove cell clumps followed by centrifugation at 300×g for 10 min at 4 °C.

#### 2.10.2. Myelin and Debris Removal

After centrifugation, cells were resuspended in a magnetically activated cell sorting (MACS) solution (0.5% BSA in PBS, pH 7.2) and incubated for 15 min at 4 °C with myelin removal beads II (Miltenyi Biotec, Cologne, Germany) for myelin and debris removal. After that, cells were washed by adding blocking solution and centrifuged at 300×g for 10 min at 4 °C. The supernatant was removed, and the pellet was resuspended in MACS solution. Then, the autoMACS^®^ Pro Separator, using a reusable autoMACS^®^ Column for separation, was prepared to isolate the cells automatically. Briefly, the tube containing the sample (row A of the rack), the tubes for collecting the labeled (myelin positive fraction; row C of the rack), and the unlabeled cell fractions (myelin negative fraction—mixed glial population, row B of the rack) were placed in the autoMACS^®^. The following program was chosen to separate these two fractions: “Depletion: Depletes—collect negative fraction in row B of the rack”.

#### 2.10.3. MACS Sorting of Adult Microglia

After myelin and debris removal, the myelin negative fraction was used to obtain the microglia. After centrifugation of the cell suspension at 300×g for 10 min at 4 °C, the cell population was resuspended in MACS solution and incubated with anti-CD11b Magnetic Microbeads (Miltenyi Biotec, Cologne, Germany) for 15 min at 4 °C. The cells were washed by adding MACS solution, and the unbound beads and debris were discarded after centrifugation at 300×g for 10 min at 4 °C. The pellets were resuspended and put in row A of the rack, and the tubes for collecting the labeled cell fractions (microglia positive fraction in row C of the rack) were placed in the autoMACS^®^ Pro Separator using the following program: “Positive selection: Possel—collect positive fraction in row C of the rack”. After centrifugation at 300×g for 10 min at 4 °C, the microglia-enriched pellets were used for RNA extraction.

### 2.11. RNA Extraction, Library Preparation, and Targeted RNA-Sequencing

The microglia-enriched pellets were resuspended in buffer RLT plus with β- mercaptoethanol for RNA extraction using the RNeasy Plus Mini Kit, along with the recommended on-column DNase digestion (Qiagen Inc., Venlo, The Netherlands). RNA quality and concentration were measured using Agilent Tech. Bioanalyzer, with samples having RNA integrity number (RIN) scores higher than 8.

The AmpliSeq Library preparation kit protocol, described in [35], was used to prepare Ion Torrent sequencing libraries. Briefly, 0.5 ng of total RNA was converted to cDNA and amplified for 16 cycles by adding PCR Master Mix and the AmpliSeq Mouse transcriptome gene expression primer pool (targeting 20,767 well-annotated RefSeq genes + 3163 XM and XR genes, based on GRCm38/mm10). The proprietary FuPa enzyme was used to digest amplicons, and then barcoded adapters were ligated onto the target amplicons. The library amplicons were bound to magnetic beads, and residual reaction components were washed off. Libraries were amplified, re-purified, and individually quantified using Agilent TapeStation High Sensitivity tape. Individual libraries were diluted to a 50 pM concentration and pooled equally, with eight individual samples (n=4 for WT and CMVMJD135 mice) per pool for further processing. Emulsion PCR, templating, and 540 chip loading were performed with an Ion Chef Instrument (ThermoFisher, Waltham, MA, USA). Ion S5XL™ sequencer (ThermoFisher, Waltham, MA, USA) was used for sequencing. Automated data analysis was performed with Torrent Suite™ Software using the Ion AmpliSeq™ RNA plugin v.5.12 and target region AmpliSeq_Mouse_Transcriptome_V1_Designed (ThermoFisher, Waltham, MA, USA).

### 2.12. Analysis of Differentially Expressed Genes and Pathways

To analyze the differentially expressed genes (DEGs), RNA expression levels were recorded as reads per million (RPM), normalized for the number of sequences reads per sample. To verify the enrichment of microglia in the samples, a list of several cell-type-specific genes was prepared [36,37,38,39,40], being described in Supplementary Data 1. A heatmap containing the cell-specific markers was achieved using the Clue Morpheus software (Broad Institute, Cambridge, MA, USA).

The Transcriptome Analysis Console (TAC) software, version 4.0.2 (Applied Biosystems by ThermoFisher, Waltham, MA, USA), was used to analyze and compare the gene expression profiles from the microglia of WT and CMVMJD135 mice. Exploratory grouping analysis was performed to identify the distribution of samples using PCA and a clustering analysis. TAC software provides the LIMMA Bioconductor package for determining differential expression based on linear models. LIMMA uses an empirical Bayes method that corrects the variance of the ANOVA analysis. Genes were considered significantly differentially expressed if they showed a |fold change|>1, p<0.05, and a false discovery rate (FDR) < 0.1. Genes overlapping between published gene sets and enriched genes in microglia of CMVMJD135 mice when compared with WT littermates were found by contingency analysis using the Fisher’s exact test and the Baptista–Pike method to calculate the odds ratio. Significance was set at p<0.05.

The TAC software and the ingenuity pathway analysis (IPA) (Qiagen Inc., Venlo, Netherlands) were used for pathways analysis. Pathways were considered significantly altered if p<0.05 and a significance value > 1.3, calculated as −log10 of the *p* value.

The validation of RNA-sequencing data was performed through quantitative RT-PCR using the same RNA used for RNA-sequencing. cDNA synthesis and quantitative RT-PCR were performed as described above. The primers were designed using NCBI Primer-BLAST and are listed in Table S3 in Supplementary Materials and Methods.

### 2.13. Statistical Analysis and Graphs

All statistical analyses were performed using the SPSS 22.0 (IBM, Armonk, NY, USA), with the GraphPad Prism 8.00 software (GraphPad Software, San Diego, CA, USA) being used to create the graphs. Regarding descriptive statistics, the mean was the considered measure of central tendency, while the extent of variability was the standard error of the mean (SEM). The normality assumption was assessed by frequency distributions (z-score of skewness and kurtosis) as well as by the Kolmogorov–Smirnov and Shapiro–Wilk tests. Levene’s test evaluated the assumption of homogeneity of variances. Most data were analyzed using the two-tailed unpaired Student’s *t*-test for comparisons between the two groups. For comparisons of more than two groups, the one-way analysis of variance (ANOVA) was used, followed by Tukey HSD or Dunnett T3’s test. Comparisons by contingency analysis used Fisher’s exact test and the Baptista–Pike method to calculate the odds ratio. The critical value for significance was set as p<0.05 throughout the study.

## 3. Results

### 3.1. Evidence of a Non-Senescent Microglial Profile in the CMVMJD135 Mouse Model of Machado–Joseph Disease

**Figure 1:**
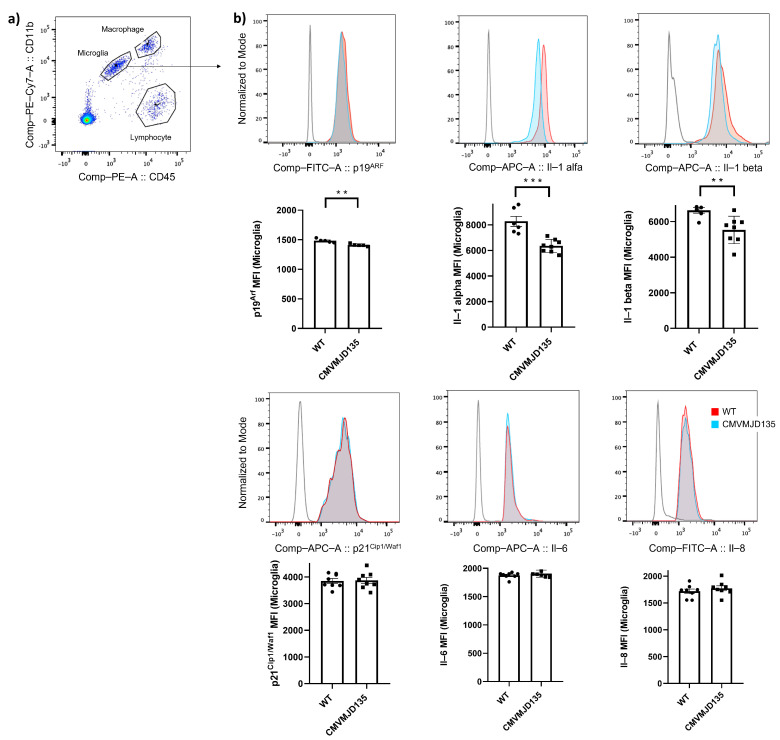
Expression of senescence markers is decreased in microglia of affected central nervous system (CNS) regions of CMVMJD135 mice. (**a**) Gating strategy used to analyze the flow cytometry data. Microglia, macrophage, and lymphocyte populations were gated using CD11b^+^CD45^mid^, CD11b^+^CD45^high^, and CD11b^low^CD45^low^, respectively; (**b**) Flow cytometry showing expression of P19^Arf^, Il-1 alpha, Il-1 beta, P21^Cip1/Waf1^, Il-6, and Il-8 in microglia (gated using CD11b^+^CD45^mid^) from wild-type (WT) and CMVMJD135 mice (n=5–8 per group). MFI = mean fluorescent intensity. Data are presented as mean + SEM (Student’s *t*-test). **, ***, represent p<0.01 and p<0.001, respectively.

Growing evidence suggests that microglia change their features with age, switching to a senescent/dystrophic profile, increasingly involved in the occurrence or aggravation, of neurodegenerative diseases [4,11,41,42]. Aging-related processes are also thought to explain the mid-life emergence of symptoms in MJD, in spite of mutant gene expression in most cell types since early development. Therefore, aging-related microglial changes could be contributing to disease onset and/or progression. To understand if microglia from CMVMJD135 mice change their features with age and switch to a senescent/dystrophic phenotype with disease progression, we evaluated protein levels of senescence markers by flow cytometry in microglia isolated from the cerebellum, brainstem, and spinal cord of these transgenic mice at 48 weeks of age (which corresponds to an advanced disease stage, Figure 1a).

Our results showed a decrease in the expression of a senescence indicator, P19^Arf^ (p=0.004549), and in the expression of senescence-associated pro-inflammatory cytokines Il-1 alpha (p=0.000416) and Il-1 beta (p=0.008074) in microglia isolated from CMVMJD135 mice compared with that of WT mice (Figure 1b). No significant differences were found in the expression of P21^Cip1/Waf1^, Il-6, and Il-8 between WT and CMVMJD135 mice (Figure 1b).

**Figure 2:**
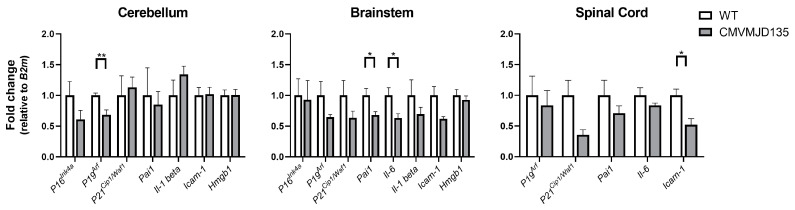
No evidence for a senescence gene expression profile in affected CNS regions of CMVMJD135 mice. The expression levels of senescence markers were analyzed in the cerebellum, brainstem, and spinal cord of 48 weeks-old WT and CMVMJD135 mice. n=4–5 per group and two technical replicates were performed. Fold change (2^−ΔΔCT^) is represented using *B2m* as a housekeeping gene. Data are presented as mean + SEM (Student’s *t*-test). *, **, represent p<0.05 and p<0.01, respectively.

To understand if a senescent profile was present in the brain of the CMVMJD135 animals, we also evaluated the mRNA expression levels of several senescence markers, including *P16^Ink4a^*, *P19^Arf^*, *P21^Cip1/Waf1^*, *Pai1*, *Il-6*, *Il-1 beta*, *Icam-1* (senescence-related intercellular adhesion molecule 1) [43], and *Hmgb1* (high mobility group box 1) [44], in whole tissue obtained from different affected regions of the CNS. In line with the results obtained by flow cytometry, we found a decrease in the expression of *P19^Arf^* (p=0.004019) in the cerebellum, a decrease in the expression of *Il-6* (p=0.031390) and *Pai1* (p=0.044628) in the brainstem, and a decrease in the expression of *Icam-1* (p=0.015626) in the spinal cord (Figure 2). However, CMVMJD135 mice displayed similar expression of *P16^Ink4a^*, *P21^Cip1/Waf1^*, *Il-1 beta*, and *Hmgb1* in the cerebellum, brainstem, and spinal cord, compared to WT mice (Figure 2). These findings indicate that microglia do not adopt a senescent-like profile in the MJD mice.

To complement our in vivo analysis, we used an experimental process, described in [11], to mimic the aging of a microglia-enriched culture (Figure S1 in Supplementary Results) and characterized the cultured cells at 4 and 16 DIV by assessing phagocytic activity and morphological changes in basal conditions or when exposed to LPS. After confirming the purity of the microglia-enriched culture obtained over time, as described in previous studies [11,45,46] (Figure S1 in Supplementary Results), and to confirm the relevance of studying cell autonomous processes in microglia in this transgenic model, we evaluated the expression levels of mutant human *ATXN3* in these cells. As expected, the expression of mutant *ATXN3* was detected in microglia from CMVMJD135 mice but not in WT mice (Figure S2 in Supplementary Results). Curiously, at 16 DIV, corresponding to the artificially “aged” microglia, no differences were found in microglia from neonatal CMVMJD135 mice upon LPS treatment (when compared to untreated cells) for all analyzed parameters (Figure S3f–i in Supplementary Results). This suggests that, with age, microglia expressing mutant *ATXN3* might show less activation in response to LPS, which could be interpreted as being indicative of senescent microglia, as dysfunctional microglia are less responsive to stimulation with age [47,48]. However, this is in contrast with our observations in the same system concerning phagocytic efficiency at 16 DIV because, like those of WT, CMVMJD135-derived microglia showed a higher phagocytic efficiency in the presence of LPS, a response that was maintained with age (Figure S4 in Supplementary Results). In addition, when analyzing the morphological changes and phagocytic efficiency among microglia derived from WT or CMVMJD135 mice, no significant differences were noted at any time point. Altogether, our in vitro results suggest that early in life, CMVMJD135-derived microglia are mostly similar to WT microglia, and that these cells do not become precociously senescent upon repeated passaging.

### 3.2. Numerical and Morphological Changes Are Observed in Microglia from CMVMJD135 Mice in a Brain Region-Dependent Manner

**Figure 3:**
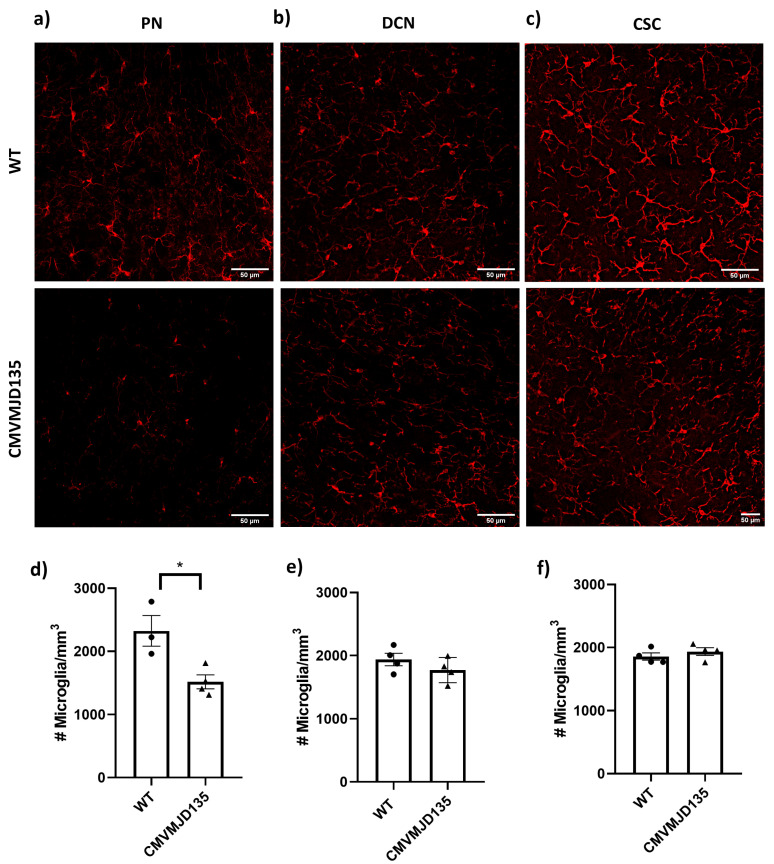
Reduction of the number of microglia in the pontine nuclei (PN) of CMVMJD135 mice. Representative images of microglial cells, using Iba-1 as a microglial marker (in red), in the (**a**) PN, (**b**) deep cerebellar nuclei (DCN), and (**c**) cervical spinal cord (CSC) of 34 weeks-old CMVMJD135 and WT mice. (**d**–**f**) Quantitative analysis of the number of Iba-1-positive cells in the PN, DCN, and CSC of WT and CMVMJD135 mice (n=4–5 per group), using ImageJ software. Data are presented as mean + SEM (Student’s *t*-test). *, represent p<0.05. Scale bar: 50 μm.

To better characterize the profile of microglia from CMVMJD135 mice, the next step was to evaluate the microglia density and morphology (Iba-1-positive cells), and to analyze their morphological features in the affected brain regions of CMVMJD135 mice, namely in the DCN, (cerebellum), the PN (brainstem), and in the cervical spinal cord (CSC), at an age when the motor phenotype of this animal model is fully established. A significant reduction in the number of microglia was found in the PN (p=0.020384) of CMVMJD135 mice compared with WT mice (Figure 3a,d). No differences were found in the DCN nor the CSC (Figure 3b,e and Figure 3c,f, respectively).

Immunostaining of the microglial marker Iba-1 was used to evaluate whether morphological alterations occur in microglia from the PN, DCN, and CSC of CMVMJD135 mice. A skeleton analysis was used to assess changes in features relevant to microglia ramification, whereas fractal analysis was used to evaluate characteristics associated with cell surface, soma thickness, cell size, the cylindrical shape of cells, the complexity of their ramifications, and the heterogeneity of their shape. The skeleton and fractal analyses showed no differences between the groups, neither in the PN nor the DCN (Figures S5–S8 in Supplementary Results).

**Figure 4:**
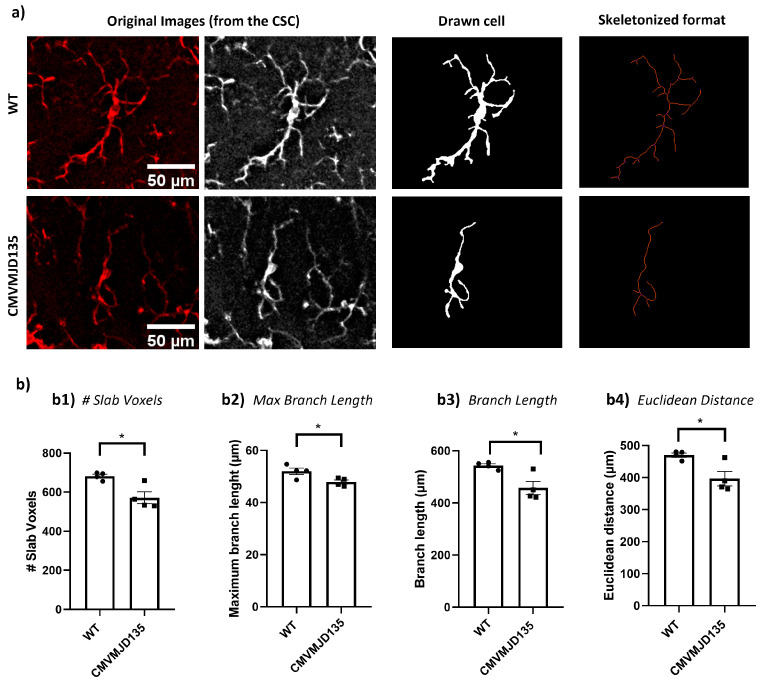
Microglia in the CSC of CMVMJD135 mice show less morphological complexity. (**a**) Representation of the process to prepare the images for skeleton analysis of microglia morphology. These images show differences regarding the number of slab voxels, the maximum branch length, the branch length, and the Euclidean distance. (**b**) Quantification of the morphometric features associated with microglia ramification, including: (**b1**) # slab voxels; (**b2**) maximum branch length; (**b3**) branch length; and (**b4**) Euclidean distance. Data of all these features were obtained from 310 microglial cells from WT mice (n=4) and 389 microglial cells from 34-week-old CMVMJD135 mice (n=4) of the CSC. Data are presented as mean + SEM (Student’s *t*-test). *, represent p<0.05. Scale bar: 50 μm.

Interestingly, the skeleton data showed significant differences in microglia from CMV-MJD135 mice in the CSC compared with WT mice. The number of slab voxels (p=0.012917), the maximum branch length (p=0.031432), the total branch length (p=0.016352), and the Euclidean distance (p=0.020316) were lower in microglia from CMVMJD135 mice (Figure 4).

**Figure 5:**
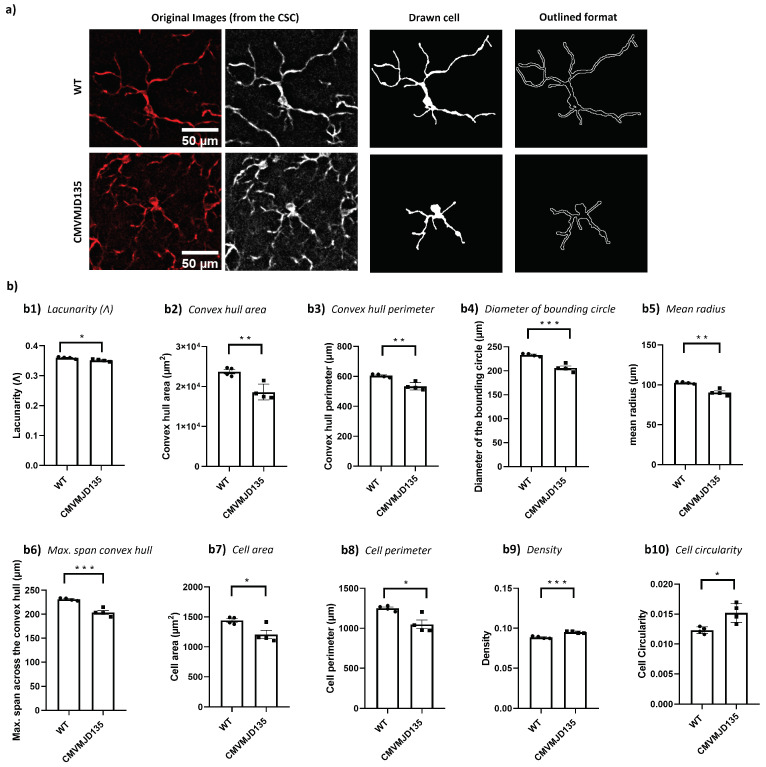
Microglia in the CSC of CMVMJD135 mice showed distinct activation-associated morphological features. (**a**) Representation of the process to prepare the images for fractal analysis of microglia morphology. These images show differences regarding the convex hull area, the mean radius, and the maximum span across the convex hull in microglia from CMVMJD135 mice. (**b**) Quantification of the morphometric features associated with heterogeneity of the shape: (**b1**) lacunarity. Associated with cell’s size: (**b2**) convex hull area, (**b3**) convex hull perimeter, (**b4**) diameter of the bounding circle, (**b5**) the mean radius, (**b6**) the maximum span across the convex hull, and (**b7**) the cell area. Associated with cell’s surface: (**b8**) cell perimeter. Associated with soma thickness: (**b9**) density and (**b10**) cell circularity. Data of all these features were obtained from 310 microglial cells from WT mice (n=4) and 389 microglial cells from 34-week-old CMVMJD135 mice (n=4) of the CSC. Data are presented as mean + SEM (Student’s *t*-test). *, **, ***, represent p<0.05, p<0.01 and p<0.001, respectively. Scale bar: 50 μm.

On the other hand, the number of branches, the number of junction voxels, the number of endpoint voxels, the average branch length, and the triple and quadruple points were similar between groups (Figure S9 in Supplementary Results). Additionally, alterations in several features associated with the heterogeneity of the shape, cell size, cell surface, and soma thickness were observed in CMVMJD135 mice. In fact, the lacunarity (p=0.017934), the convex hull area (p=0.003983), the convex hull perimeter (p=0.001963), the diameter of the bounding circle (p=0.000753), the mean radius (p=0.001132), the maximum span across the convex hull (p=0.000757), the cell area (p=0.021343), and the cell perimeter (p=0.011744) were found to be decreased in microglia from CMVMJD135 mice, whereas density (p=0.000798) and cell circularity (p=0.014008) were increased in this group (Figure 5).

Regarding the features associated with the complexity of ramifications and with the cylindrical shape of the cells, no differences were observed between groups (Figure S10 in Supplementary Results). These observations suggest that microglia in the CSC of CMVMJD135 mice are more activated when compared with WT mice since these microglia have fewer and shorter branches, with smaller size and higher soma thickness.

### 3.3. Euclidean Distance, Convex Hull Area, Mean Radius, and Maximum Span across the Convex Hull Are the Features That Best Characterize Spinal Cord Microglia of MJD Mice

**Figure 6:**
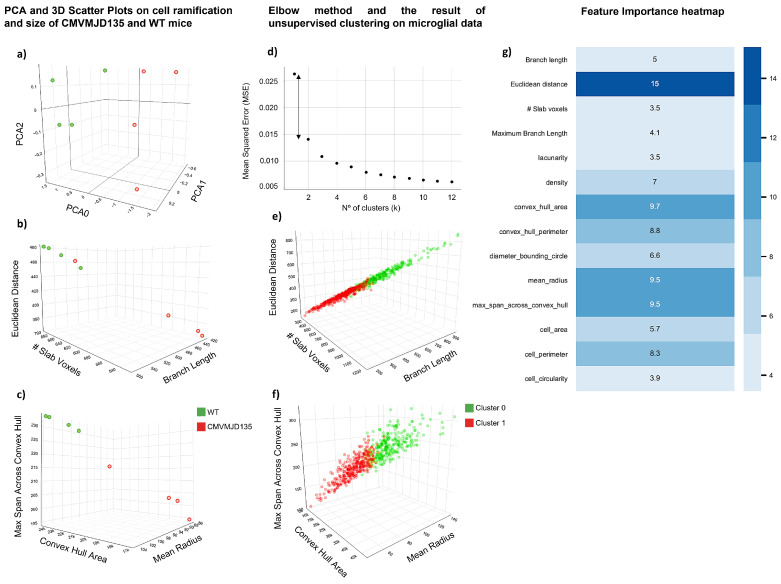
A clear separation of microglia in the CSC of CMVMJD135 and WT mice regarding features associated with cell ramification and cell size. (**a**) A 3D scatter plot showing the distribution of CMVMJD135 mice (in red) and WT animals (in green) on a principal components plane. (**b**) A 3D scatter plot showing a clear separation between CMVMJD135 and WT mice regarding the number of slab voxels, branch length, and Euclidean distance; and (**c**) a 3D scatter plot showing a clear separation between CMVMJD135 and WT mice regarding their convex hull area, mean radius, and maximum span across the convex hull. (**d**) Graphical result of the elbow method applied on the dataset comprised of 310 cells from WT mice and 389 from CMVMJD135 ones. (**e**,**f**) Data points of a total of 310 microglial cells from WT mice and 389 microglial cells from CMVMJD135 mice were plotted as a function of the significant features, belonging to one of two clusters: cluster 0, in green, or cluster 1, in red. (**e**) A 3D scatter plot showing the relationship between the number of slab voxels, branch length, and Euclidean distance; and (**f**) a 3D scatter plot showing the relationship between the convex hull area, mean radius, and maximum span across the convex hull of all microglia. (**g**) Feature importance heatmap for each parameter used to classify microglia from CMVMJD135 and WT mice. The higher the color tone, the higher the importance of the parameter.

Since our initial analysis revealed changes in microglia in the spinal cord, a region that is affected since early stages in MJD patients and in the CMVMJD135 mouse model, PCA and machine learning models were implemented to further characterize the morphological changes between CMVMJD135- and WT-derived microglia, allowing the identification of potential clusters of cells based on their morphological features and pinpointing those features that assume higher importance. A morphological analysis of microglia from the CSC of CMVMJD135 and WT mice was performed by measuring a total of 25 different features related to microglia ramification, complexity, and cell shape. Regarding microglial ramification features, four were statistically different in microglia from CMVMJD135 mice (Figure 4). On the other hand, from the 15 features associated with complexity and cell shape, 10 were found to be significantly different between the groups (Figure 5). Considering the number of significantly altered features, a PCA was performed to reduce this dimensionality. A 3D space was computed based on three principal components, the PCA being able to preserve 99.1% of all information present in the 14 significant features (PC0 = 91.7%, PC1 = 5.8%, and PC2 = 1.6%; Figure 6a). A scatter plot was designed, plotting each animal as a point in a 3D space on the principal components plane. Figure 6a depicts a clear separation between CMVMJD135 and WT animals, based on the three principal components that are grounded on the statistically different features. The exception was one CMVMJD135 mouse, which was closer to the WT group. To better visualize the relationships between multiple significant features found to be altered between both groups, two more 3D scatter plots were conceived. Figure 6b,c shows a clear distinction between the two groups based on cell ramification and cell size features, respectively. Both plots show a clear distinction between CMVMJD135 and WT animals based on the morphological features of their microglia.

With the PCA showing promising prospects, an unsupervised machine learning model, the k-means, was used to identify clusters of data with similar characteristics. The used dataset comprised 310 microglial cells from the CSC of WT mice and 389 from the CSC of CMVMJD135 ones. Using all 14 statistically significant features, the elbow method, as depicted in Figure 6d, identified the largest drop in the error for *k* = 2, i.e., identified two clusters in the dataset, which is in accordance with the expectations since these data originated from two groups (CMVMJD135 and WT). Once the ideal number of clusters was found, these clusters were plotted in a 4D space, with color as the fourth dimension. One cluster, in green, groups more ramified cells, with longer branches, larger area and perimeter, and lower circularity and density. This cluster is mainly composed of microglia from WT mice. Conversely, the second cluster, in red, is primarily composed of CMVMJD135 microglia, which have fewer and shorter branches, smaller size, and higher soma thickness, characteristics typically found in activated microglia (Figure 6e,f).

To complement this analysis, gradient boosted trees were conceived, optimized, and evaluated, the goal being to use a machine learning model that is able to distinguish microglia from CMVMJD135 and WT mice, in the CSC. Four independent trials were run, using nested k-fold cross-validation (5 outer and 5 inner folds). While the input data are all the significant features, the label was set as the parameter identifying WT and CMVMJD135 cells, this being a binary classification problem. The candidate models were tuned regarding the number of estimators, learning rate, tree’s max depth, and fraction of columns to be sub-sampled, being evaluated by its accuracy, precision, and recall. The best candidate model attained an accuracy of approximately 70% using one-fifth of the total number of columns per estimator, 100 estimators, a tree’s max depth of 2, and a learning rate of 0.1. Since gradient boosted trees provide the ability to obtain estimates of feature importance, Figure 6g depicts the importance attained by each feature. While the machine learning model allocates lower importance to features such as the cell’s lacunarity and circularity, and the number of slab voxels, features such as the Euclidean distance, convex hull area, mean radius, and maximum span across the convex hull have increased importance when identifying microglia based on their morphological features. This further reinforces the significance of these morphological features to characterize spinal cord microglia of MJD mice, denoting the impact of the disease in these morphological characteristics.

### 3.4. Transcriptomic Profiling of Microglia in the Pathogenesis of MJD

To further explore the molecular profile of MJD-associated microglia, RNA-sequencing analysis was performed on microglia isolated from WT and CMVMJD135 animals at 34 weeks of age. The analysis of the transcriptomic data confirmed that specific markers for microglia were expressed at high levels. In contrast, other cell-type markers were expressed at shallow levels, indicating that the microglial samples from CMVMJD135 and WT mice presented high purity (Figure S11a–d in Supplementary Results), even though a residual expression of some oligodendrocyte-specific genes was found (Figure S11e in Supplementary Results).

**Figure 7:**
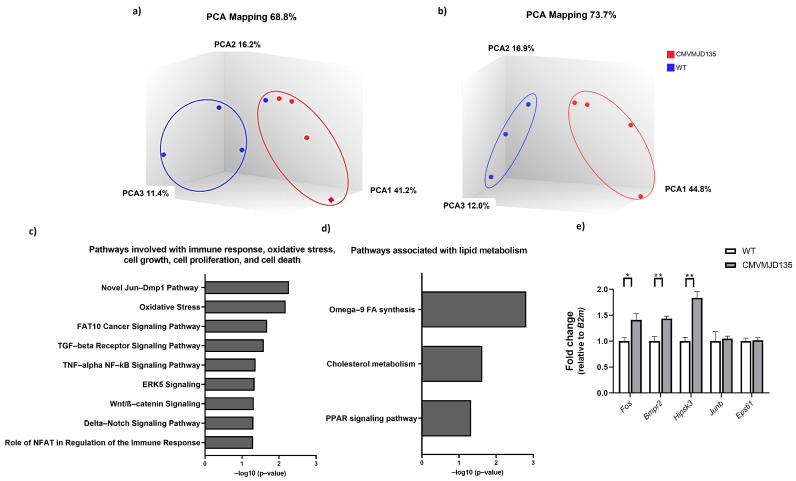
Up-regulated differentially expressed genes (DEGs) found in CMVMJD135-derived microglia are associated with immune response, oxidative stress, cell growth, cell proliferation, cell death, and lipid metabolism pathways. Before the analysis of the DEGs, and of the molecular pathways altered, a principal components analysis (PCA) was conceived to evaluate if CMVMJD135 and WT mice showed distinct profiles. (**a**) The PCA sets one WT sample within the vicinity of the CMVMJD135 cluster. WT cluster presents a sparser configuration. (**b**) PCA shows a clear expression separation between CMVMJD135 and WT without sample WT1. WT cluster presents a denser configuration. Three biological replicates for WT mice and four biological replicates for CMVMJD135 mice. Pathways significantly altered were found in microglia from CMVMJD135 mice compared with WT mice. (**c**) Pathways associated with immune response, oxidative stress, cell growth, cell proliferation, and cell death. (**d**) Pathways associated with lipid metabolism. All pathways are presented in descending order of significance. (**e**) Expression analysis performed on the selected genes confirmed the results obtained from RNA-sequencing analysis of microglia. An increase in the expression of *Fos*, *Bmpr2*, and *Hipsk3* was found in microglia from CMVMJD135 mice. n=3–4 per group, and two technical replicates were performed. Fold change (2^−ΔΔCT^) is represented using *B2m* as a housekeeping gene. Data are presented as mean + SEM (Student’s *t*-test). *, **, represent p<0.05 and p<0.01, respectively.

The PCA and hierarchical clustering heatmap confirmed that CMVMJD135 and WT mice showed distinct transcriptional profiles (Figure 7a,b), revealing a non-overlapping clustering of samples in each group, with the exception of one sample from the WT group, the PCA being able to preserve 68.8% of all information (PC1 = 41.2%, PC2 = 16.2%, and PC3 = 11.4%). This WT outlier, which overlapped with samples of CMVMJD135 mice instead of WT (Figure 7a), was discarded from the analysis to remove overlapping clusters, thus improving the amount of information captured by the PCA, which rose to 73.7% (PC1 = 44.8%, PC2 = 16.9%, and PC3 = 12.0%). Figure 7b depicts the non-overlapping clusters of samples in each group, indicating a distinct profile among genotypes.

The TAC software then determined the number of DEGs in microglia from CMV-MJD135 mice. In total, 101 DEGs were identified: 83 up-regulated and 18 down-regulated genes. The complete list of DEGs is provided in Figure S12 in Supplementary Results.

### 3.5. Transcriptional Changes Seen in CMVMJD135 Microglia Overlap Those in Amyotrophic Lateral Sclerosis and Alzheimer Disease Mouse Models

Next, we compared the list of transcripts found to be differentially expressed in CMVMJD135 mice with 40 different datasets of previously reported DEGs, which include, among others, data on the microglial signature program [49,50] on other neurodegenerative disorders [51,52,53,54,55,56,57,58,59,60,61,62], aging [38,51,63], disease-associated microglia (DAM) [64], and injury-related microglia [38,51]. We found a significant overlap with only 3 of the 40 published gene sets, namely with the DEGs seen in microglia of a mouse model of amyotrophic lateral sclerosis (ALS), the SOD1^G93A^ mouse model [52]; of a mouse model of Alzheimer disease (AD), the App^NL-G-F/NL-G-F^ mouse model [52]; and with a list of microglial genes highly expressed and/or affected in different neuroinflammatory conditions [65] (Figure S13 in Supplementary Results and Supplementary Data 2).

The SOD1^G93A^ mouse model of ALS shared 27 deregulated genes with CMVMJD135 mice. Of these 27 overlapping genes, 17 displayed a similarly altered gene expression profile: *Lamc1*, *Hipk3*, *Lrrc58*, *Bmpr2*, *Nav1*, *St8sia4*, *Cpd*, *Fmnl2*, *Atp6v0a1*, *Klhl24*, *Cnot1*, *Tmem106b*, *Xpr1*, and *Rnh1* are up-regulated in both SOD1^G93A^ and CMVMJD135 mice; and *Bend6*, *Ups11*, and *Tbkbp1* are down-regulated in both models. However, *Ncam1*, *Arhgef15*, *Abcb1a*, *Alpl*, *Foxf2*, *Caskin2*, *Fbxl12*, *Gpld1*, and *Csad* genes were found to be up-regulated in CMVMJD135 mice but down-regulated in SOD1^G93A^ mice. In contrast, the *Plin2* gene was found to be down-regulated in CMVMJD135 mice but up-regulated in SOD1^G93A^ mice (Figure S13 in Supplementary Results and Supplementary Data 2).

Most of the genes (19 out of 31) that showed an overlap with the App^NL-G-F/NL-G-F^ mouse model of AD were discordant regarding their altered gene expression profile. In fact, while that in CMVMJD135 mice the *Cux2*, *Ncam1*, *Arhgef12*, *Mkl2*, *Arhgef15*, *Abcb1a*, *Tyro3*, *Alpl*, *Foxf2*, *Sox8*, *Ahnak*, *Caskin2*, *Scd2*, *Atp2b4*, *Sh2d5*, *Gpld1*, and *Syt3* genes were found to be up-regulated and the *Fbxw4* and *Plin2* genes were down-regulated, in App^NL-G-F/NL-G-F^ mice, the same genes were found to be down-regulated and up-regulated, respectively. Regarding the remaining overlapping genes, some were found to be up-regulated in both App^NL-G-F/NL-G-F^ and CMVMJD135 mice (*Gm6548*, *Rnf144b*, *Epsti1*, *St8sia4*, *Cpd*, *Fos*, *Junb*, *Acsl4*, and *Klhl24*), while others were found to be down-regulated in both models (*Bend6*, *Phlpp1*, and *Rbfox1*). We also found a positive association of two CMVMJD135-altered genes with the cluster of microglial genes highly expressed in neuroinflammatory conditions. In particular, *Mefc2* and *Fos*, two of the up-regulated genes found in CMVMJD135-derived microglia, were implicated in neuroinflammation conditions [65] (Figure S13 in Supplementary Results and Supplementary Data 2).

Overall, these results suggest a path of disease with higher similarity to that of ALS, a motor neuron disease, than with that of AD and other more “neuroinflammatory diseases”.

### 3.6. Up-Regulated DEGs Found in CMVMJD135-Derived Microglia Are Associated with Immune Response, Oxidative Stress, Cell Growth, Cell Proliferation, Cell Death, and Lipid Metabolism Pathways

An analysis of the involvement of the DEGs found in CMVMJD135-derived microglia in different biological pathways was performed. This analysis revealed eight DEGs associated with cellular processes, such as immune response, oxidative stress, cell growth, cell proliferation, and cell death. The pathways found to be significantly altered in microglia from CMVMJD135 mice when compared with WT mice were as follows: oxidative stress (*Junb* and *Fos* (also known as *c-Fos*)); TGF-β receptor signaling pathway (*Fos*, *Junb*, and *Mef2c*); TNF-α NF-kβ signaling pathway (*Gsk3β*, *Usp11*, and *Alpl*); role of NFAT in regulation of the immune response (*Fos*, *Gsk3β*, and *Mef2c*); the novel Jun-Dmp1 pathway (*Junb* and *Fos*); FAT10 cancer signaling pathway (*Bmpr2* and *Gsk3β*); ERK5 signaling (*Fos* and *Mef2c*); Wnt/β-catenin signaling (*Bmpr2*, *Gsk3β*, and *Sox8*); and delta-notch signaling pathway (*Gsk3β* and *Mef2c*). All the indicated genes showed increased expression in microglia from CMVMJD135 mice, except for *Usp11*, which showed decreased expression (Figure 7c).

Interestingly, the altered gene expression also suggested changes in the microglial lipid metabolism. These include the Omega-9 FA synthesis pathway, cholesterol metabolism (consists of both Bloch and Kandutsch–Russell pathways), and PPAR signaling pathway. The *Acsl4* and *Scd2* DEGs were found to be involved in these lipid metabolism pathways (Figure 7d). It was also found that the expression of genes related to oxidative stress, particularly the synthesis of nitric oxide (NO), was increased in CMVMJD135 mice, as seen by the up-regulation of *Gsk3β*, *Junb*, *Cpd*, *Igfbp3*, and *Ntn1*.

The RNA-sequencing results were further validated through qPCR. Five DEGs—*Fos*, *Junb*, *Bmpr2*, *Hipsk3*, and *Epsti1*—were validated with acceptable cycle threshold (CT) values. While no statistically significant differences were found in the expression of *Junb* and *Epsti1*, the results were similar to those obtained by RNA-sequencing, with an increase in the expression of *Fos* (p=0.019), *Bmpr2* (p=0.006), and *Hipsk3* (p=0.003) in microglia from CMVMJD135 mice (Figure 7e).

## 4. Discussion

The contribution of microglia to several neurodegenerative diseases is well recognized, and these cells play a pivotal role in their pathogenesis, often with different contributions at different disease stages and in distinct brain regions [10,13,66]. Microglial subpopulations called DAM have been defined for several neurodegenerative diseases, chronic neuroinflammatory states, and aging [52,64,67]. Yet, little is known about the profile of microglia and their involvement in MJD. In this study, we characterized the profile of microglia in a mouse model of MJD, with a particular focus on the brainstem, cerebellum, and spinal cord, three of the CNS areas most affected in this polyglutamine disease [21,22].

Because MJD pathophysiology appears gradually and progresses over time [21,22], and microglia were described to become senescent/dystrophic in other neurological disorders, including AD, Parkinson’s disease (PD), multiple sclerosis (MS), Huntington’s disease (HD), and ALS [4,11,41,42,68,69], we first set out to investigate if microglia from CMVMJD135 mice displayed an accelerated senescence profile. For this, the typical signs of cellular senescence were further evaluated in brain microglia from CMVMJD135 mice.

The senescence phenotype is associated with an increased expression of specific proteins, considered senescence indicators, including some cell cycle regulators and senescence-associated pro-inflammatory cytokines [15]. Through these, the so-called senescence-associated secretory phenotype (SASP) may generate an inflammatory environment and induce senescence in neighbor cells, which may exert a deleterious effect and promote neuron degeneration [68]. Contrary to what is described in the literature for other neurodegenerative disorders [4,15,67,68], our observations showed a decrease in the protein levels of a senescence indicator, P19^Arf^ and of senescence-associated pro-inflammatory cytokines Il-1 alpha and Il-1 beta in microglia from CMVMJD135 mice when compared to those of WT animals. This was consistent with the results of our analysis of senescence-related genes in whole tissue from three affected regions of MJD mice, in which we found a decrease in the expression of *P19^Arf^* in the cerebellum, of *Il-6* and *Pai1* in the brainstem, and of *Icam-1* in the spinal cord. Overall, our data do not support a significant contribution of cell senescence processes (in microglia or other cell types) to MJD, even at late stages.

The characterization of morphological changes of microglia from CMVMJD135 mice was also performed in affected CNS regions at a late disease stage. Since dystrophic cells can display some of the features typically associated with activation, mostly de-ramification and shortening of the processes, it is difficult to distinguish, with certainty, “activated” from “dystrophic” microglia [70]. However, other abnormal morphological features, such as gnarled, beaded, unusually tortuous, or fragmented cytoplasmic processes, are usual signs of senescent microglia [4,42,71,72]. These allow us to distinguish between an “activated state”, which is characterized by ramified cells with a larger cell body and shorter, thick processes, and a “reactive state”, typically characterized by smaller, spherical cells, which can also display amoeboid-like morphologies [6,7]. These microglial states, which display inflammatory and phagocytic features, are most often observed in pathological situations [7]. However, in some neurological conditions, and depending on the stage of the pathological process, microglia can play both a toxic or a protective role. Hence, the extent of microglial activation and, thereby, their contribution to the pathogenesis may depend on the type and duration of injury [6,11,45,67,73,74] and on the CNS region under study [67]. A better comprehension of MJD-associated microglia based on the characterization of their morphological profile may help to unravel the relevance of these cells in MJD pathogenesis.

Of the three analyzed regions, only microglia from the spinal cord (one of the earliest affected CNS regions in this mouse model) showed significant differences in features associated with ramification, heterogeneity of the shape, cell size, cell surface, and soma thickness. Indeed, microglia from CMVMJD135 mice showed a decreased number of slab voxels, a decrease in the maximum branch length and branch length, and lower Euclidean distance, which is an indicator of the cell’s tortuosity [75]. Thus, these microglia are less ramified, with shorter processes, and less tortuous when compared with microglia from WT mice. In addition, we found an increased density and circularity of microglia from CMVMJD135 mice. As described in [9], circularity determines the cell’s roundness, which is increased in amoeboid-like cells. On the other hand, an increased density occurs during the morphological shift from a ramified to an amoeboid shape upon neuroinflammatory insults, a phenotype seen upon exposure to stress [8]. Features associated with cell size, such as convex hull area, convex hull perimeter, diameter of the bounding circle, the convex hull area, the mean radius, the maximum span across the convex hull, and the cell area, were lower in microglia from CMVMJD135 mice. Previous studies show that decreased values of such features are associated with amoeboid-like shapes [6,7,9]. Finally, the lacunarity, which refers to the degree of inhomogeneity, was found to be decreased in microglia from CMVMJD135 mice, implying that these cells have a more homogeneous outline when compared with cells from WT mice [9]. These results were complemented with the PCA and machine learning models outcome, which depicted a clear structure on the morphological data, with two clusters being identified. While one is mainly composed of WT-derived microglia (more ramified cells, with longer branches, larger area and perimeter, and lower circularity and density), the other mainly groups CMVMJD135-derived microglia (with fewer and shorter branches, smaller size, and higher soma thickness). The supervised machine learning model, which was tuned to identify the cells’ genotype based on their morphological features, allowed us to further identify those that best characterize spinal cord microglia of MJD mice (Euclidean distance, convex hull area, mean radius, and maximum span across the convex hull), these being the features that are most affected by this disease. Overall, these observations are particularly relevant and may indicate that microglia in the spinal cord of CMVMJD135 mice are more activated than WT-derived microglia. Even though the morphological changes point to an increased activation state of microglia, and other studies show microgliosis in MJD patients [23,24,25] and MJD mice [26], further mechanistic studies are required to understand whether these microglial cells actively contribute to MJD onset and/or progression.

The RNA-sequencing analysis on microglia isolated from the cerebellum and brainstem (as a whole), of WT and CMVMJD135 animals, identified significantly altered genes and molecular pathways in CMVMJD135 mice. From the 101 DEGs found in CMVMJD135-derived microglia, 8 (*Junb*, *Fos*, *Bmpr2*, *Gsk3β*, *Mef2c*, *Usp11*, *Alpl*, and *Sox8*) were found to be overlapping several significantly altered pathways related to the immune response, oxidative stress, cell growth, cell proliferation, and cell death. Other cellular pathways were also changed, namely, some associated with lipid metabolism.

In a mouse model of ALS, the microglial transcriptional factor *c-Fos* was significantly down-regulated. This alteration is associated with restoring the abnormal microglial phenotype and attenuation of the disease [76]. While some studies show that *c-Fos* suppresses the expression of pro-inflammatory phenotype-associated genes, such as inducible NO synthase (*iNOS*) [77], tumor necrosis factor-alpha (*Tnfα*), and *Il-6* through the suppression of NF-kβ activity [78], suggesting that it acts as an anti-inflammatory transcription factor essential for microglia survival [76,78], other studies show that the blockade of c-Fos with dexmedetomidine halts microglia inflammation and inhibits postoperative cognitive dysfunction in AD patients, thus setting c-Fos as a potential anti-inflammatory therapeutic target for NDs [79]. Regarding the Gsk3β, its activation is associated with increased neuroinflammation and microglial activation. Some studies have demonstrated that Gsk3β promotes microglial responses to inflammation, and that the use of Gsk3β inhibitors, such as lithium, SB216763, kenpaullone, and indirubin-3’-monoxime, provides a means to limit the inflammatory actions of microglia and provides protection from inflammation-induced neuronal toxicity [80]. Another study reinforces Gsk3β-mediated neuroinflammation, partially by enhancing nuclear factor kappa b subunit 1 (Nfkb1) signaling, where the inhibition of Gsk3β with the SB216763 inhibitor reduces Nfkb1 signaling and inflammation levels, in a mouse model of Rett syndrome [81]. The expression of the *BMPR2* gene by microglia is scarcely referred to in the literature. Still, it is increased in active multiple sclerosis lesions, suggesting a possible role for this gene in MS pathogenesis [82]. Regarding the *Alpl* gene encoding the Alkaline phosphatase, tissue-nonspecific isozyme protein, known to have a role in brain development and function [83], it was demonstrated that its activity is increased in both brain and plasma of AD patients, inducing neuronal toxicity via tau dephosphorylation [84,85]. On the other hand, the transcription factor *Mef2c* was reported to be expressed in both mouse and human microglia and is known to be involved in microglial specification [86,87]. Moreover, decreased function of Mef2c is associated with a possible microglial activation that is sufficient to induce autism-like symptoms in mice [88]. Additionally, *Mef2c* normally restrains the microglial inflammatory response, and its expression is lost in aged brains in a type I interferon (IFN-I)-dependent manner [89]. These facts demonstrate that the activity of Mef2c becomes critical under pathological conditions and with aging, when the levels of inflammatory cytokines are increased. The *Usp11* gene, on the other hand, was demonstrated to regulate microglial activation and neuroinflammation in intracerebral hemorrhage (ICH). Thus, silencing *Usp11* was put forward as a novel anti-inflammatory method for ICH treatment since it blocks the release of pro-inflammatory cytokines by microglia, leading to protection from neurological impairment [90]. Hence, the decreased expression of *Usp11* in the brain of MJD mice could indicate a similar adaptive and protective response.

The role of lipid metabolism in the polarization of microglial inflammatory status was recently explored and may inspire novel approaches that modulate metabolism to ameliorate neuroinflammatory and NDs [91,92,93,94]. In fact, regarding the specific MJD DEGs here identified and known to be involved in the lipid metabolism, *Acsl4* was found to be a novel regulator of neuroinflammation in ischemic stroke, and the knockdown of *Acsl4* expression was proposed to provide a potential therapeutic target through the inhibition of pro-inflammatory cytokine production in microglia [95]. Meanwhile, the *Scd2* gene was found to be down-regulated upon activation of microglia induced by LPS [96].

The expression of genes related to the synthesis of nitric oxide (NO) was found to be increased in microglia from CMVMJD135 mice, namely of *Gsk3β*, *Junb*, *Cpd*, *Igfbp3*, and *Ntn1*. This pathway is known to be implicated in the pathogenesis of NDs, in which elevated NO provokes either neuroinflammation or apoptosis in microglia [97]. As mentioned above, Gsk3β and Junb are associated with increased neuroinflammation and microglial activation [80,81,98]. However, an increase in *Igfbp3* expression was seen in an ischemic injury mouse model to lead to increased microglial apoptosis and to a reduction of activated microglia. These findings imply that *Igfbp3* can act as an anti-inflammatory factor [99]. In addition, Ntn1 was put forward as a novel therapeutic agent to ameliorate early brain injury via its anti-inflammation effect, by suppression of microglia activation, peroxisome proliferator-activated receptor (PPARγ) activation, inhibition of factor nuclear kappa β (NF-kβ), and decrease in Tnfα, Il-6, and Icam-1 [100].

Interestingly, we also found multiple deregulated genes that are common in both CMVMJD135-derived microglia and microglia of the neurodegenerative mouse models of ALS and AD. However, while some of them displayed a similarly altered gene expression profile, others were discordant. To the best of our knowledge, apart from nine genes (*Atp6v0a1*, *Tmem106b*, *Bmpr2*, *Ups11*, *Fos*, *Junb*, *Acsl4*, *Tyro3*, and *Scd2*), the overlapping of the remaining 49 genes with datasets of DEGs from neurodegenerative mouse models of ALS and AD, is here reported for the first time. Only three remain to be described from the nine genes identified above (*Atp6v0a1*, *Tmem106b*, and *Tyro3*). Regarding the *Atp6v0a1* gene, it was found that the attenuation of the human microglial inflammation and suppression of the expression *IL-1 beta* and *IL-6* by the increase in *ATP6V0A1* expression with rifampicin improved the lysosomal function, which may be a novel therapeutic strategy for PD [101]. The *TMEM106B* gene was found to be involved in the pathological processes of AD, whose expression is reduced in AD brains [102]. On the other hand, it was demonstrated that the TAM (Tyro3, Axl, Mer) family of receptor tyrosine kinases limit inflammatory responses upon Toll-like receptors stimulation in microglia, with a positive impact on AD progression [103]. Another study reported that the loss of TAM receptors affects adult brain neurogenesis, which was attributed to exaggerated inflammatory responses by microglia characterized by increased mitogen-activated protein kinases (MAPK) and NF-kβ activation, as well as to an increased production of pro-inflammatory cytokines [104].

As described above, we found genes (such as the *Fos*, *Junb*, *Gsk3β*, *Acsl4*, and *Bmpr2*) that, when up-regulated, promote pro-inflammatory microglial responses. The use of inhibitors of these genes and the proteins they encode may provide a means to offer protection from inflammation-induced neuronal toxicity, i.e., these genes could be potential targets to counteract MJD. However, we also found genes (such as the *Mefc2*, *Scd2*, *Igfbp3*, *Ntn1*, *Usp11*, *Atp6v0a1*, and *Tyro3*) that promote the inhibition of inflammation in microglia through the inhibition of pro-inflammatory cytokine production, which could correspond to an endogenous neuroprotective response and explain the decrease in expression of genes encoding pro-inflammatory cytokines, such as *Il-6*, *Il-1 alpha*, *Il-1 beta*, and *Icam-1* in CMVMJD135 mice. Overall, the profile of MJD microglia is mixed regarding pro- and anti-inflammatory molecule expression, and the overlapping results suggest a higher similarity of MJD with ALS than AD, which is not unexpected, given the shared involvement of motor systems in these two disorders. Furthermore, since microgliosis was observed in MJD patients’ post-mortem brains [23,24,25], it would be interesting to explore, in future studies, whether the genes and pathways identified in the CMVMJD135 mouse model of MJD are also altered in the brains of MJD patients, at the mRNA or protein level.

To the best of our knowledge, this is the first study to characterize the functional and morphological features of microglia in an in vivo model of MJD and to provide new insights into the transcriptomic profile of these cells in the context of this disorder. While no evidence for senescence of microglia or other brain cells was found in the CMVMJD135 mouse model, our findings revealed morphological alterations in microglia from the spinal cord of these mice, which point to an increased activation state of these cells when compared with those of WT animals. In addition, the conceived supervised machine learning model revealed key morphological features that are most affected by the disease, with the possibility of using such features to distinguish between CMVMJD135- and WT-derived microglia. Finally, the results obtained from the transcriptomic analysis provided the identification of molecular pathways that may constitute potential targets to counteract this disease, and suggest that, among others, the lipid metabolism should be further investigated in these cells.

## Data Availability

The data presented in this study are available on request from the corresponding author.

## Supplementary Materials

The following supporting information can be downloaded at: https://www.mdpi.com/article/10.3390/biomedicines10020237/s1: Supplementary Data 1—lists of cell-type-specific genes. Supplementary Data 2—Genes overlapping between published gene sets and enriched genes in CMVMJD135 and wild-type (WT)-derived microglia. Supplementary Materials and Methods—Table S1. Organization of the experimental groups to evaluate microglia phagocytic ability and morphology in culture; Table S2. List of primary and secondary antibodies used in flow cytometry and immunofluorescence; Table S3. List of primers used in reverse-transcription quantitative real-time PCR; Figure S1. The process to prepare binary (black and white) images for fractal and skeleton analysis; Figure S2. The *MorphData* and *AnalyzeSkeleton 2D/3D* plugins applied to the skeletonized images; Figure S3. The outline images were processed using the *MorphData* and *FracLac* plugins. Supplementary Results—Figure S1. Confirmation of the high purity of microglia in culture; Figure S2. Expression of mutant *ATXN3* in microglia from CMVMJD135 mice at two different time points in culture; Figure S3. Microglia expressing *ATXN3* showed a less activated phenotype in response to lipopolysaccharides (LPS) in artificially “aged” primary cultures; Figure S4. CMVMJD135 and Wild-Type (WT)-derived microglia showed an increased phagocytic efficiency in the presence of LPS in culture; Figure S5. The ramification state of microglia in the pontine nuclei (PN) of the CMVMJD135 mice is similar to those of microglia from WT mice; Figure S6. The complexity and shape of microglia in the PN of CMVMJD135 mice are similar to those of microglia from WT mice; Figure S7. Microglia in the deep cerebellar nuclei (DCN) of CMVMJD135 mice showed no differences in features relevant to microglia ramification; Figure S8. Microglia in the DCN of CMVMJD135 mice showed no changes in the complexity and shape; Figure S9. Some parameters associated with microglia ramification were similar between CMVMJD135 and WT mice in the cervical spinal cord (CSC); Figure S10. No changes were observed in the parameters related to the complexity of ramifications and with the cylindrical shape of the cells between groups in the CSC; Figure S11. Evaluation of microglial enrichment in RNA-sequencing samples; Figure S12. Differential gene expression between microglia from CMVMJD135 and WT mice; Figure S13. Transcriptional changes seen in CMVMJD135 microglia overlap those in amyotrophic lateral sclerosis (ALS) and Alzheimer’s disease (AD) mouse models.
