# Supplementary material for: Profiling Microglia in a Mouse Model of Machado–Joseph Disease
Source: Biomedicines. 2022 Jan 23;10(2):237. doi: 10.3390/biomedicines10020237 (PMC8869404; doi:10.3390/biomedicines10020237)
Supplement: Supplementary file 1 [file biomedicines-10-00237-s001.zip › Supplementary_Materials_and_Methods.pdf]

Supplementary Information of the Materials and Methods

“Profiling microglia in a mouse model of Machado-Joseph disease”

by Campos et al., 2022

Contents within the present file:

1. **Supplementary methods**
   - **1.1** Primary culture of microglial cells.
   - **1.2** Purity assessment of microglia culture over time.
   - **1.3** Image analysis for microglial density and morphological analysis.

2. **Supplementary Tables:**

   Suppl. Table S1. Organization of the experimental groups to evaluate microglia phagocytic ability and morphology in culture.

   Suppl. Table S2. List of primary and secondary antibodies used in flow cytometry and immunofluorescence.

   Suppl. Table S3. List of primers used in reverse-transcription quantitative real-time PCR.

3. **Supplementary Figures:**

   Suppl. Figure S1. The process to prepare binary (black and white) images for fractal and skeleton analysis.

   Suppl. Figure S2. The *MorphData* and *Skeleton 2D/3D* plugins applied to the skeletonized images.

   Suppl. Figure S3. The outline images were processed using the *MorphData* and *FracLac* plugins.

4. **Supplementary References**

1. Supplementary methods

1.1 Primary culture of microglial cells

Primary mixed glial cultures, composed of astrocytes and microglia, were prepared from brainstem and cerebellum together of CMVMJD135 and Wild-Type (WT) neonatal mice (3-4 days old). Replicating the same methods described in [11], cells (4×10^5 cells/cm^2) were plated on uncoated 12-well tissue culture plates (with 18 mm coverslips) in culture medium (DMEM F12 GlutaMAX-I supplemented with 10 % fetal bovine serum (FBS) and 1 % antibiotic-antimycotic solution) and kept at 37 ºC in a humidified atmosphere of 5 % CO_2. Medium was replaced every 3 days and maximum confluency and purity of the cultures were achieved after 21 days in mixed culture.

Microglia-enriched culture were obtained from both WT and CMVMJD135 mice by mild trypsinization, as previously described in [45]. Concisely, mild trypsinization was performed with a trypsin-EDTA solution diluted 1:3 in DMEM F12 GlutaMAX-I for 45-60 min, which promoted the detachment of an upper layer of cells containing all the astrocytes, while microglia remained at the bottom of the well. The initial mixed glia-conditioned medium was added after the removal of the medium containing the detached cells.

1.2 Purity assessment of microglia culture over time

Before proceeding to the characterization of microglia from WT and CMVMJD135 mice over time, the purity of microglia in culture was evaluated using the cells from WT neonatal mice. Briefly, after the mild trypsinization, the purity of these cultures was assessed by immunocytochemistry, in three different time points (4, 10, and 16 days *in vitro* (DIV)), using the anti-ionized calcium binding adaptor molecule 1 (Iba-1) antibody as a microglial marker, and anti-glial fibrillary acid protein (GFAP) antibody as an astrocyte marker, followed by species-specific fluorescent-labelled secondary antibodies, Alexa Fluor 594 goat anti-rabbit and Alexa Fluor 488 goat anti-mouse, respectively (Supplementary Table S2 in *Supplementary Materials and Methods*). Cells were then incubated with the 4',6-Diamidin-2-phenylindol (DAPI, Invitrogen) diluted 1:1000 in Phosphate Saline Buffer (PBS). After washing the cells, the coverslips were mounted on microscope Superfrost©Plus slides using an aqueous mounting medium.

11 to 33 random fluorescence images were acquired in each coverslip using Olympus Widefield Inverted Microscope IX81 with a resolution of 1024×1024 px and a 20× objective. At least 3 coverslips per each independent experiment (n=3-4) in each time point (4,10, and 16 DIV)

were used for counting the number of microglia and astrocyte cells. Total count of Iba-1-positive cells and GFAP-positive cells was obtained using the Point Tool feature of ImageJ software. Quantification was then normalized to the total photo area (624.39×624.39 µm).

To induce reactivity in CMVMJD135 and WT -derived microglia, 100 ng/mL of LPS was added to the culture medium for 24 h before each time point. Microglial cultures of two experimental groups, WT and CMVMJD135, in two different conditions, basal or exposed to LPS, at two different time points, 4 and 16 DIV, were studied as presented in Supplementary Table S1 in *Supplementary Materials and Methods*.

1.3 Image analysis for microglial density and morphological analysis

To obtain fractal and skeleton data, binary images (white cells on black background) are required [32]. Hence, after stacking the 3D volume images, the double-color image was split to obtain the Iba-1 label in the red channel, and brightness and contrast were adjusted. The despeckle filter was then used to remove salt and pepper noise, with the threshold option being used and adjusted, as needed. At least 5 cells from both the original and the binary images were selected with the rectangle tool, using the region of interest (ROI) to set the same rectangle dimensions for all the selected cells. After selecting the cells, the paintbrush tool was used to complete and draw the morphology of the cells (always comparing them with the original ones) and to clean extra signal that is not related to these cells, thus producing a single-cell image without any noise. Finally, the binary single-cell was converted into an outline or skeletonized format for fractal or skeleton analysis, respectively [32] (Supplementary Figure S1 in *Supplementary Materials and Methods*).

The analyze *skeleton 2D/3D* plugin (developed by and maintained here: https://imagej.net/plugins/analyze-skeleton), was applied to binary single-cells, tagging skeletal features relevant to microglia ramification: number of endpoints voxels (#/cell), number of junctions voxels (#/cell), number of slab voxels (#/cell), number of branches (#/cell), number of triple points (#/cell), number of quadruple points (#/cell), Euclidean distance (µm/cell), branch length (µm/cell), average branch length (µm/cell), and maximum branch length (µm/cell) (Supplementary Figure S2 in *Supplementary Materials and Methods*).

A fractal analysis was carried out using the *FracLac* plugin (Karperien A., FracLac for ImageJ (https://imagej.nih.gov/ij/plugins/fraclac/FLHelp/Introduction.htm)) setting the Num G option to 4 and checking the metrics box. Then, the scan was run to obtain the hull and circle results, selecting only the data of interest associated with the cell's surface (cell perimeter and roughness), the soma thickness (cell circularity and density), the cell's size (mean radius, convex hull perimeter, convex hull circularity, bounding circle diameter, maximum span across the convex hull, convex hull area, and cell area), and the cylindrical shape of the cells (convex hull span ratio

and the ratio of convex hull radii). Data from the box count summary were also obtained, being associated with the complexity of ramifications (fractal dimension - D) and the heterogeneity of the shape (lacunarity - Λ) (Supplementary Figure S3 in *Supplementary Materials and Methods*).

2. Supplementary Tables

Supplementary Table S1. Organization of the experimental groups to evaluate microglia phagocytic ability and morphology in culture.

	4 DIV				16 DIV			
	**WT**		**CMVMJD135**		**WT**		**CMVMJD135**	
Condition	Basal	LPS	Basal	LPS	Basal	LPS	Basal	LPS
Nº Animals	3	3	5	5	4	4	3	3
Nº of Cells	387	309	597	543	504	318	279	351
Coverslips per animal	3-4				3-4			
Images per coverslip	7-22				7-22			

Supplementary Table S2. List of primary and secondary antibodies used in flow cytometry and immunofluorescence.

Antibodies	Supplier	Dilution	Technique
Primary antibodies			
PE anti-mouse CD45 antibody	BioLegend<br>Catalog nº 103106	1:200	Flow cytometry
PE/Cy7 anti-mouse/human CD11b antibody	BioLegend<br>Catalog nº Cat#101215	1:200	Flow cytometry
Alexa Fluor 647 anti-mouse/human CD11b antibody	BioLegend<br>Catalog nº 101218	1:200	Flow cytometry
Anti-rat CDKN2A/p19ARF antibody	Santa Cruz biotechnology<br>Catalog nº sc -32748	1:200	Flow cytometry
Anti-rabbit Cdkn1a/p21Cip1/Waf1 antibody	Abcam<br>Catalog nº ab188224	1:50	Flow cytometry
Anti-mouse CXCL8/IL-8 antibody	GeneTex<br>Catalog nº GTX15763	2μg/ml	Flow cytometry
Anti-rabbit IL-6 antibody	Cell Signalling<br>Catalog nº #12912	1:400	Flow cytometry
Anti-rabbit IL-1 beta antibody	Abcam<br>Catalog nº ab9722	1:100	Flow cytometry
Anti-rabbit IL-1 alpha antibody	Abcam<br>Catalog nº ab7632	1:100	Flow cytometry
Anti-rabbit Iba-1 antibody	Wako<br>Catalog nº 019-19741	1:600	Immunofluorescence
Anti-mouse GFAP antibody	EMD Millipore<br>Catalog nº MAB360	1:500	Immunofluorescence
Secondary antibodies			
Goat Anti-Rabbit IgG (H+L) Cross-Adsorbed Secondary Antibody, Alexa Fluor 594	Thermo Fisher Scientific<br>Catalog nº # A-11012	1:1000	Immunofluorescence
Goat Anti-mouse IgG (H+L) Cross-Adsorbed Secondary Antibody, Alexa Fluor 488	Thermo Fisher Scientific<br>Catalog nº # A-11001	1:1000	Immunofluorescence
Goat anti-Rabbit IgG (H+L) Cross-Adsorbed Secondary Antibody, Alexa Fluor 647	Thermo Fisher Scientific<br>Catalog nº #A-21244	1:1000	Flow cytometry
Goat anti-mouse IgG (H+L) Cross-Adsorbed Secondary Antibody, Alexa Fluor 488	Thermo Fisher Scientific<br>Catalog nº #A-11001	1:1000	Flow cytometry
Goat anti-rat IgG (H+L) Cross-Adsorbed Secondary Antibody, Alexa Fluor 488	Thermo Fisher Scientific<br>Catalog nº #A-11006	1:1000	Flow cytometry

Supplementary Table S3. List of primers used in reverse-transcription quantitative real-time PCR**.**

Gene	Sequence		Reference
	Forward (5´to 3´)	Reverse (5´to 3´)	
*p16^INK4a^*	5’- CCCAACGCCCCGAACT- 3’	5’- GCAGAAGAGCTGCTACGTGAA- 3’	[14]
*p19^Arf^*	5’- AGGCCGGCAAATGATCATAGA - 3’	5’- ACTTCCAAACATCATGAC CTGC - 3’	Primer-BLAST
*p21^Cip1/Waf1^*	5’- GTCCAATCCTGGTGATGTCC - 3’	5’- GTTTTCGGCCCTGAGATGT - 3’	[14]
Pai1	5’- GCACTGCAAAAGGTCAGGAT - 3’	5’- TGGCCCATGAAGAGGATTGT - 3’	[14]
Icam-1	5’- CCATCACCGTGTATTCGTTTC - 3’	5’- AGGTCCTTGCCTACTTGCT - 3’	Primer-BLAST
Hmgb1	5’- CCATTGGTGATGTTGCAAAG - 3’	5’- CTTTTTCGCTGCATCAGGTT - 3’	Primer-BLAST
Il-1 beta	5’- ACCTTCCAGGATGAGGACATGA - 3’	5’- AACGTCACACACCAGCAGGTTA - 3’	Primer-BLAST
Il-6	5’- ACACATGTTCTCTGG GAAATCGT- 3’	5’-AAGTGCATCATCGTTGTTCATACA - 3’	Primer-BLAST
Fos	5’- ATGGTGAAGACCGTGTCAGG - 3’	5’- GTTGATCTGTCTCCGCTTGGA - 3’	Primer-BLAST
Bmpr2	5’- GAGCACAGAGGCCCAATTCT - 3’	5’- ATCTTGTGTTGACTCACCTATCTGT - 3’	Primer-BLAST
Hipk3	5’- AGAAAGCGGGTGTGAGACTG - 3’	5’- GGCTGGCATGTAGAATCCGT - 3’	Primer-BLAST
Junb	5’- AGGCAGCTACTTTTCGGGTC - 3’	5’- TTGCTGTTGGGGACGATCAA - 3’	Primer-BLAST
Epsti1	5’- CGAGAGCATCATCAGTCCAAAAC - 3’	5’- GTCCATCCCTCGTCTTTTGC - 3’	Primer-BLAST
human ataxin-3	5’- GGAACAATGCGTCGGTTG - 3’	5’- GCCCTAACTTTAGACATGTTAC - 3’	[29]
mouse ataxin-3	5’- TGTCTTGTTACAGAAAGATCAG - 3’	5’- GTTACAAGAACAGAGCTGACT - 3’	[29]
B2m	5’- CCTTCAGCAAGGACTGGTCT - 3’	5’- TCTCGATCCCAGTAGACGGT - 3’	Primer-BLAST

3. Supplementary Figures

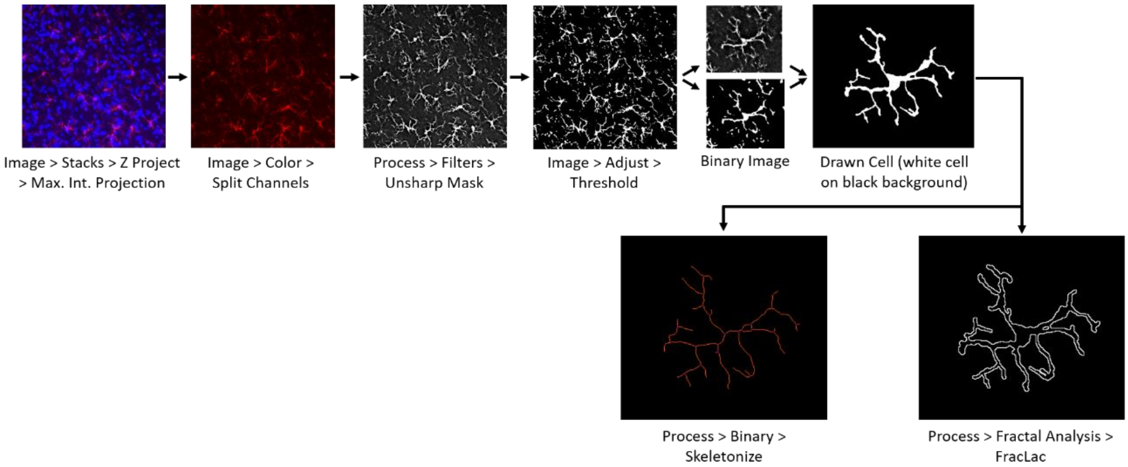

Supplementary Figure S1. The process to prepare binary (black and white) images for fractal and skeleton analysis. Several steps were followed to apply commands and options to obtain single-cell binary of microglia from original Z-stacks images with double-color image (Iba-1 labels the microglia in the red channel and DAPI labels the nuclei in the blue channel). Binary single-cells were then converted into an outline or skeletonized format for fractal or skeleton analysis, respectively. Adapted from [32].

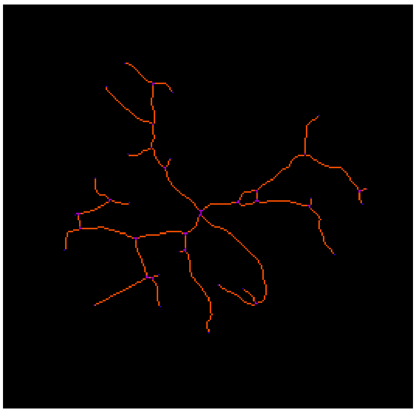

Cell Ramification

Supplementary Figure S2. The *MorphData* and *Skeleton 2D/3D* plugins were applied to the skeletonized images, which tag skeletal features relevant to microglia ramification: branch length and slab voxels as orange, endpoints as blue, and junctions as purple. Adapted from [32].

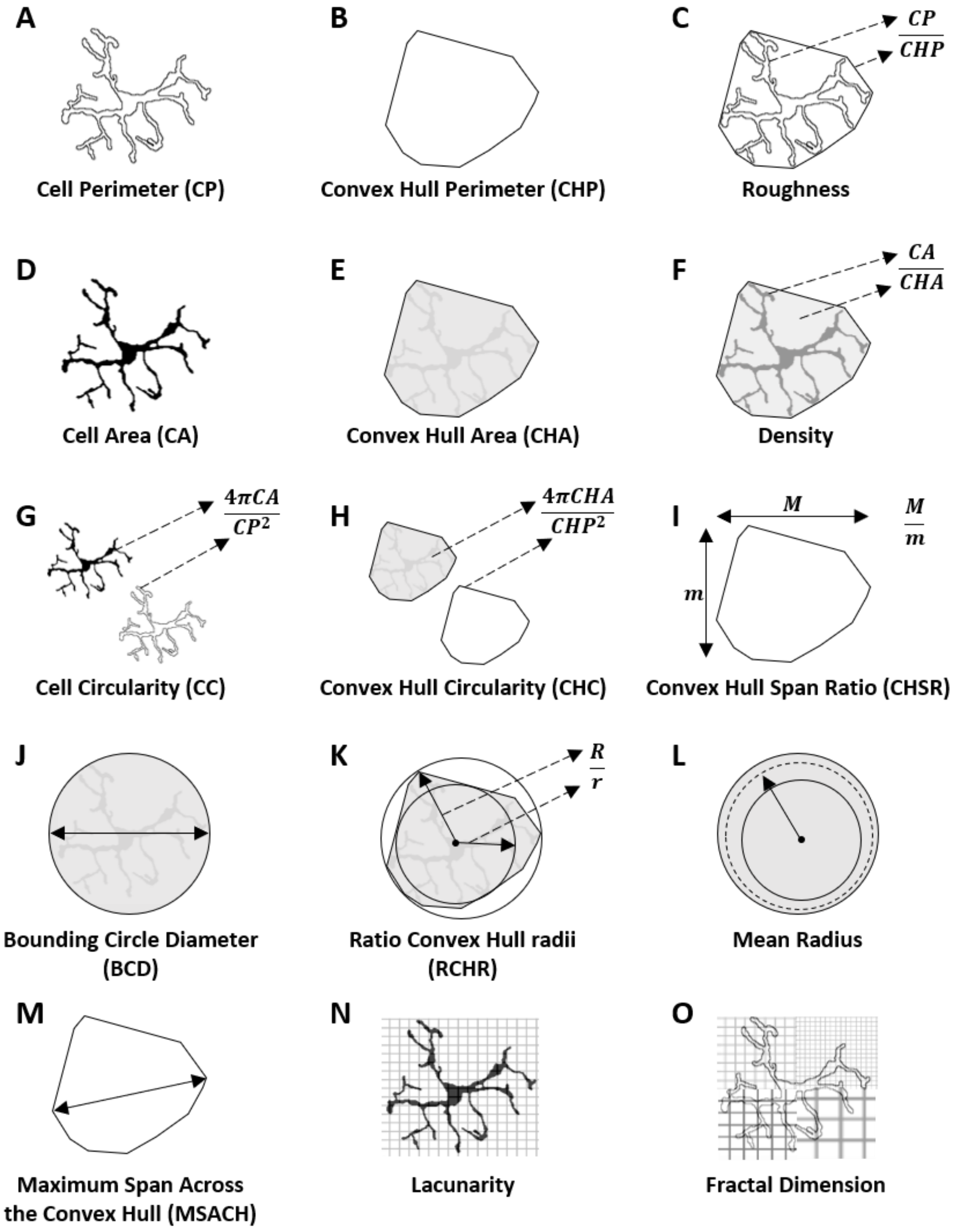

Supplementary Figure S3. The outline images were processed using the *MorphData* and *FracLac* plugins to obtain data regarding the hull and circle results such as **(a)** cell perimeter, **(b)** convex hull perimeter, **(c)** roughness, **(d)** cell area, **(e)** convex hull area, **(f)** density, **(g)** cell circularity, **(h)** convex hull circularity, **(i)** convex hull span ratio, **(j)** bounding circle diameter, **(k)** ratio convex hull radii, **(l)** mean radius, and **(m)** maximum span across the convex hull. Regarding the box count summary, **(n)** lacunarity and **(o)** fractal dimension were the obtained data. Adapted from [6].

4. Supplementary References

[6] Fernandez-Arjona, M.D.M.; Grondona, J.M.; Fernandez-Llebrez, P.; Lopez-Avalos, M.D. Microglial Morphometric Parameters Correlate With the Expression Level of IL-1β, and Allow Identifying Different Activated Morphotypes. Frontiers in Cellular Neuroscience 2019, 13. doi:10.3389/fncel.2019.00472.

[11] Caldeira, C.; Oliveira, A.F.; Cunha, C.; Vaz, A.R.; Falcao, A.S.; Fernandes, A.; Brites, D. Microglia change from a reactive to na age-like phenotype with the time in culture. Frontiers in Cellular Neuroscience 2014, 8. doi:10.3389/fncel.2014.00152.

[14] Bussian, T.J.; Aziz, A.; Meyer, C.F.; Swenson, B.L.; van Deursen, J.M.; Baker, D.J. Clearance of senescent glial cells prevents tau-dependent pathology and cognitive decline. Nature 2018, 562, 578–582. doi:10.1038/s41586-018-0543-y.

[29] Silva-Fernandes, A.; Costa, M.C.; Duarte-Silva, S.; Oliveira, P.; Botelho, C.M.; Martins, L.; Mariz, J.A.; Ferreira, T.; Ribeiro, F.; Correia-Neves, M.; Costa, C.; Maciel, P. Motor uncoordination and neuropathology in a transgenic mouse model of Machado–Joseph disease lacking intranuclear inclusions and ataxin-3 cleavage products. Neurobiology of Disease 2010, 40, 163–176. doi:10.1016/j.nbd.2010.05.021.

[32] Young, K.; Morrison, H. Quantifying Microglia Morphology from Photomicrographs of Immunohistochemistry Prepared Tissue Using ImageJ. Journal of Visualized Experiments 2018, 136. doi:10.3791/57648.

[45] Saura, J.; Tusell, J.M.; Serratosa, J. High-yield isolation of murine microglia by mild trypsinization. Glia 2003, 44, 183–9. doi:10.1002/glia.10274.
