# Supplementary material for: Profiling Microglia in a Mouse Model of Machado–Joseph Disease
Source: Biomedicines. 2022 Jan 23;10(2):237. doi: 10.3390/biomedicines10020237 (PMC8869404; doi:10.3390/biomedicines10020237)
Supplement: Supplementary file 1 [file biomedicines-10-00237-s001.zip › Supplementary_Results.pdf]

Supplementary Data of the Results

"Profiling microglia in a mouse model of Machado-Joseph disease"

by Campos et al., 2022

Contents within the present file:

1. **Supplementary Figures:**

Suppl. Figure S1. Confirmation of the high purity of microglia in culture.

Suppl. Figure S2. Expression of mutant *ATXN3* in microglia from CMVMJD135 mice at two different time points in culture.

Suppl. Figure S3. Microglia expressing *ATXN3* showed a less activated phenotype in response to lipopolysaccharides (LPS) in artificially "aged" primary cultures.

Suppl. Figure S4. CMVMJD135 and Wild-Type (WT)-derived microglia showed an increased phagocytic efficiency in the presence of LPS in culture.

Suppl. Figure S5. The ramification state of microglia in the pontine nuclei (PN) of the CMVMJD135 mice is similar to those of microglia from WT mice.

Suppl. Figure S6. The complexity and shape of microglia in the PN of CMVMJD135 mice are similar to those of microglia from WT mice.

Suppl. Figure S7. Microglia in the deep cerebellar nuclei (DCN) of CMVMJD135 mice showed no differences in features relevant to microglia ramification.

Suppl. Figure S8. Microglia in the DCN of CMVMJD135 mice showed no changes in the complexity and shape.

Suppl. Figure S9. Some parameters associated with microglia ramification were similar between CMVMJD135 and WT mice in the cervical spinal cord (CSC).

Suppl. Figure S10. No changes were observed in the parameters related to the complexity of ramifications and with the cylindrical shape of the cells between groups in the CSC.

Suppl. Figure S11. Evaluation of microglial enrichment in RNA-sequencing samples.

Suppl. Figure S12. Differential gene expression between microglia from CMVMJD135 and WT mice.

Suppl. Figure S13. Transcriptional changes seen in CMVMJD135 microglia overlap those in Amyotrophic lateral sclerosis (ALS) and Alzheimer disease (AD) mouse models.

2. Supplementary References

Purity assessment of microglia culture over time

a)

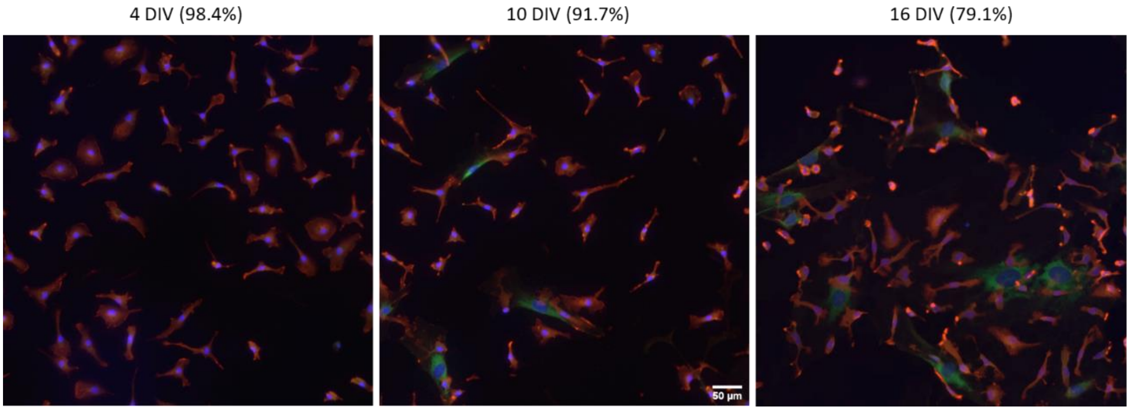

b)

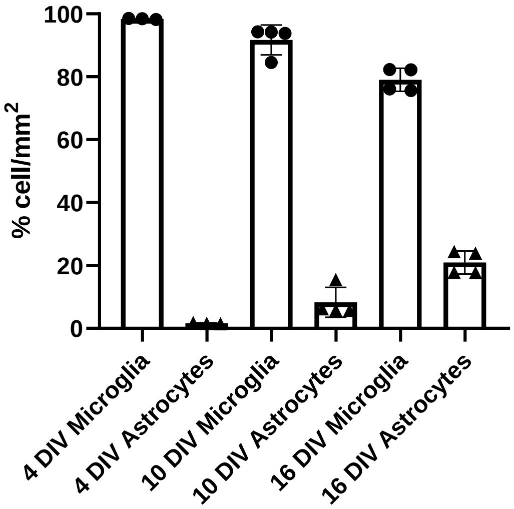

Supplementary Figure S1. **Purity assessment of microglia culture over time. (a)** Representative images of immunocytochemistry using Iba-1 as a microglial marker (in red) and glial fibrillary acid protein (GFAP) as an astrocyte marker (in green) over time. **(b)** At 4 days *in vitro* (DIV), a high purity was observed (98%), with a slight contamination with astrocytes occurring over time, but maintaining a 79% purity at 16 DIV. N = 3-4 independent experiments per each time point (4, 10, and 16 DIV). Each value represents the mean ± SEM. Scale bar: 50 µm.

Evaluation of the expression levels of mutant human *ATXN3* in microglia

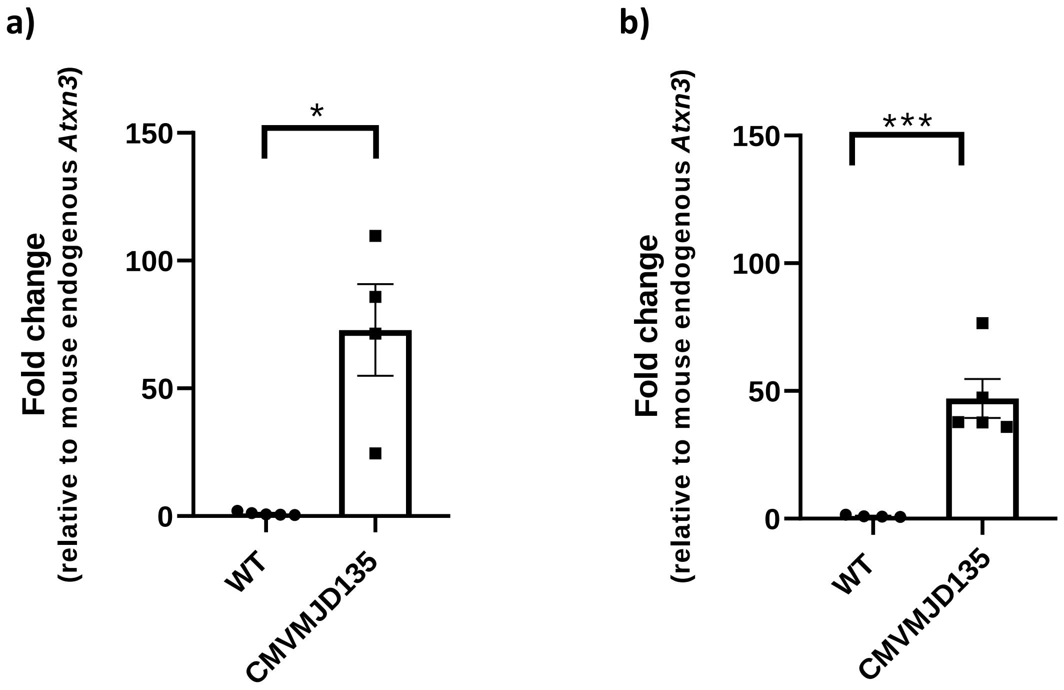

Supplementary Figure S2. Expression of mutant *ATXN3* in microglia from CMVMJD135 mice at two different time points in culture. a) at 4 DIV, and b) at 16 DIV. Cultures of n = 4-5 animals per group. Two technical replicates were performed. Fold change (2-ΔΔCT) is represented using mouse endogenous *Atxn3* as reference gene. Data are presented as mean+SEM (Student's t-test). *, ***, represent $p < 0.05$ and $p < 0.001$, respectively.

Evaluation of microglial morphology in culture

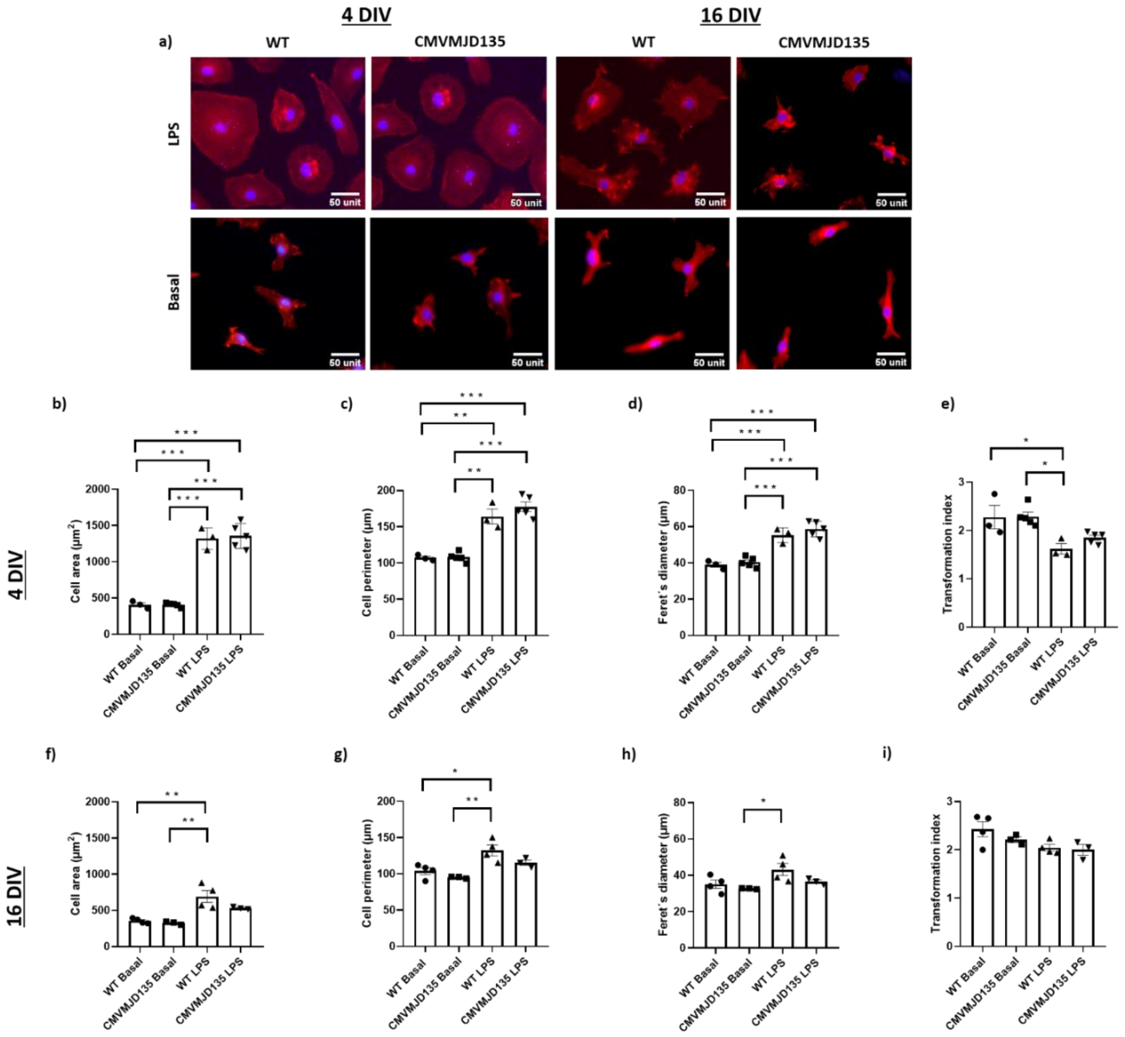

Supplementary Figure S3. Microglia expressing *ATXN3* showed a less activated phenotype in response to lipopolysaccharides (LPS) in artificially "aged" primary cultures. **a)** Images that represent the morphological changes of microglia, as observed by immunocytochemistry using microglia-specific marker Iba-1 (in red), from CMVMJD135 and Wild-Type (WT) mice, in the absence/presence of LPS, over time, in culture; **b)** cell area; **c)** cell perimeter; **d)** Feret's diameter; and **e)** transformation index. **b-e)** measured at 4 DIV; and **f-i)** measured at 16 DIV. Cultures of n = 3-5 animals per group. Data are presented as mean+SEM, (One-way ANOVA (Post hoc Tukey's test)). *, **, ***, represent $p < 0.05$, $p < 0.01$ and $p < 0.001$, respectively. Scale bar unit as μm.

Evaluation of microglia phagocytic ability in culture

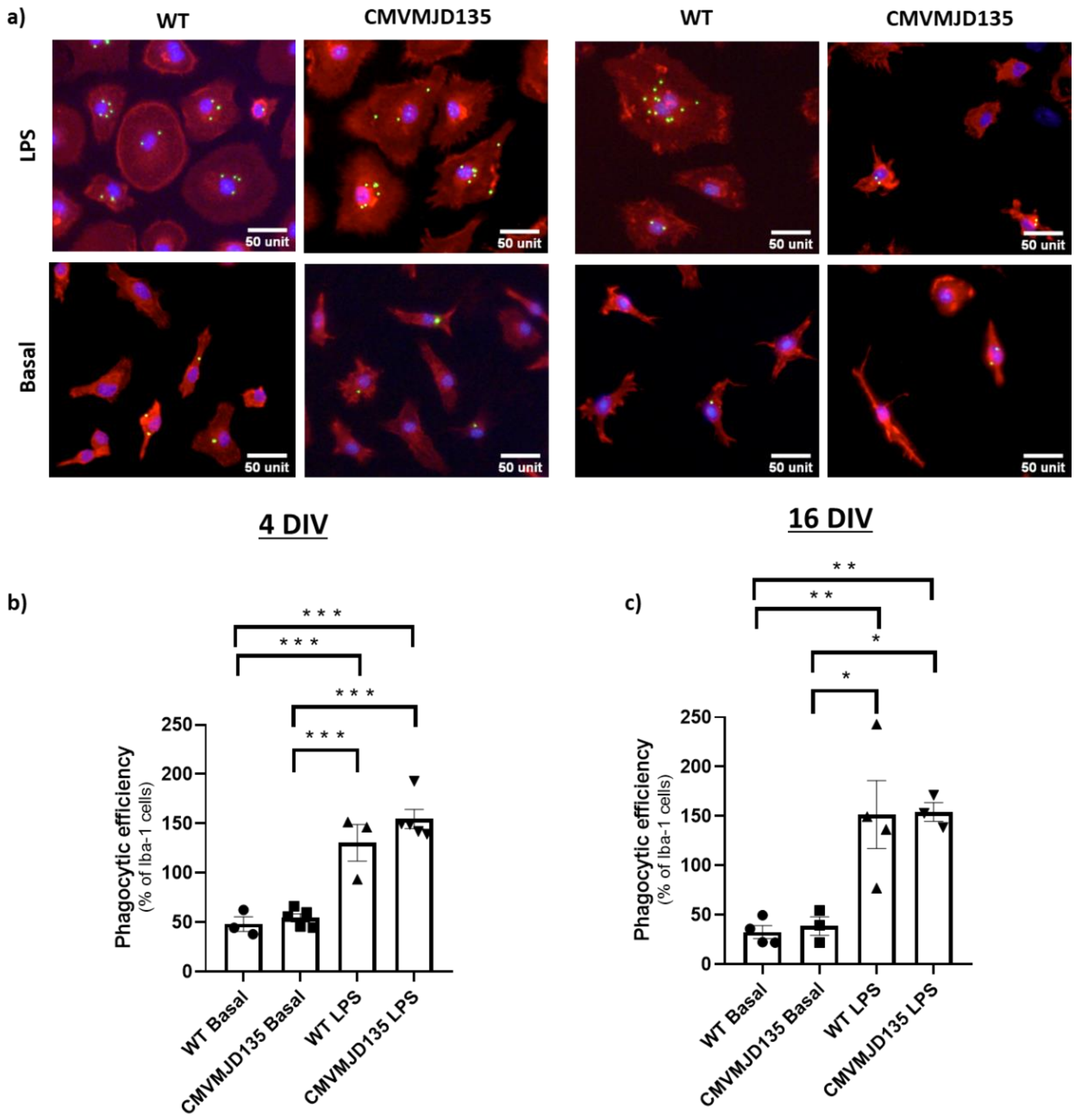

Supplementary Figure S4. CMVMJD135 and WT-derived microglia showed an increased phagocytic efficiency in the presence of LPS in culture. **a)** Representative images of the phagocytic capacity of CMVMJD135 and WT-derived microglia immunostained for Iba-1 (in red) and counterstained with 4',6-Diamidin-2-phenylindol (DAPI) for nuclei staining (in blue) containing phagocytosed fluorescent beads (in green), in the absence/presence of LPS, over time in culture. **b, c)** Phagocytic efficiency (%) was measured using ImageJ and calculated as previously described. Cultures of n = 3-5 per group. Data are presented as mean+SEM, (One-way ANOVA (Post hoc Tukey's test)). *, **, ***, represent $p < 0.05$, $p < 0.01$ and $p < 0.001$, respectively. Scale bar unit as µm.

.

<u>Morphological analysis of microglia from the PN of CMVMJD135 and WT mice</u>

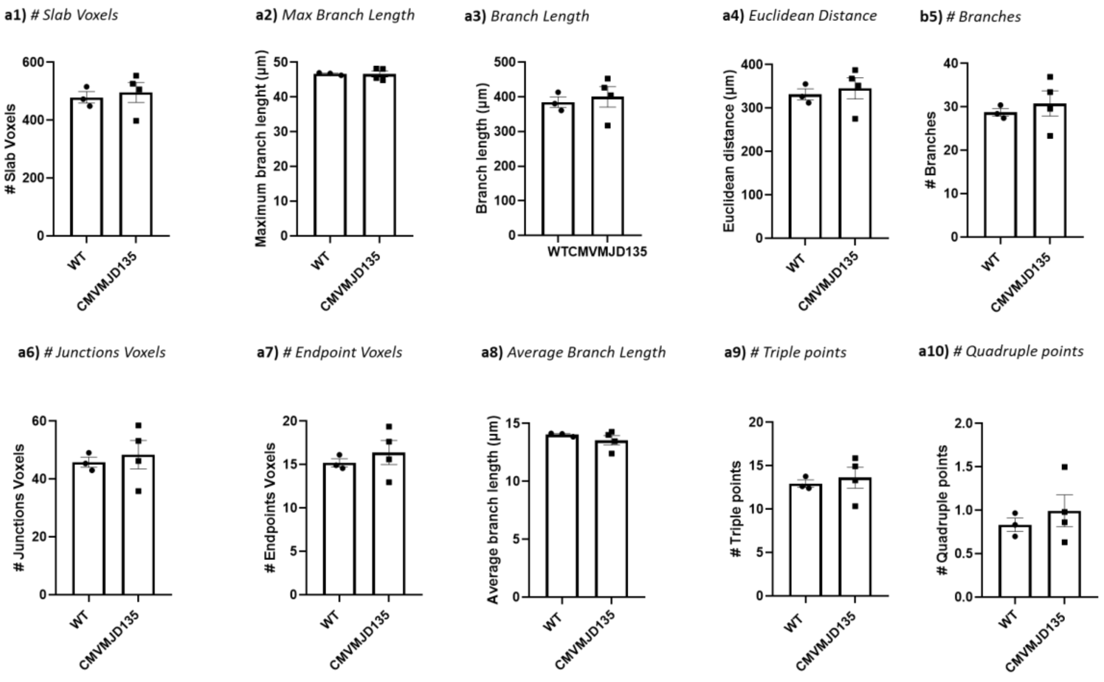

Supplementary Figure S5. The ramification state of microglia in the pontine nuclei (PN) of the CMVMJD135 mice is similar to those of microglia from WT mice. a) Quantification of the morphometric parameters associated with microglia ramification, including: **a1)** # slab voxels; **a2)** maximum branch length**; a3)** branch length; **a4)** Euclidean distance; **a5)** # branches**; a6)** # junctions' voxels; **a7)** # endpoints voxels; **a8)** average branch length; **a9)** # triple points**;** and **a10)** # quadruple points. Values for all these parameters were obtained from 152 microglial cells from WT mice (n = 3) and 180 microglial cells from CMVMJD135 mice (n = 4) of the PN. Data are presented as mean+SEM (Student's t-test).

Morphological analysis of microglia from the PN of CMVMJD135 and WT mice

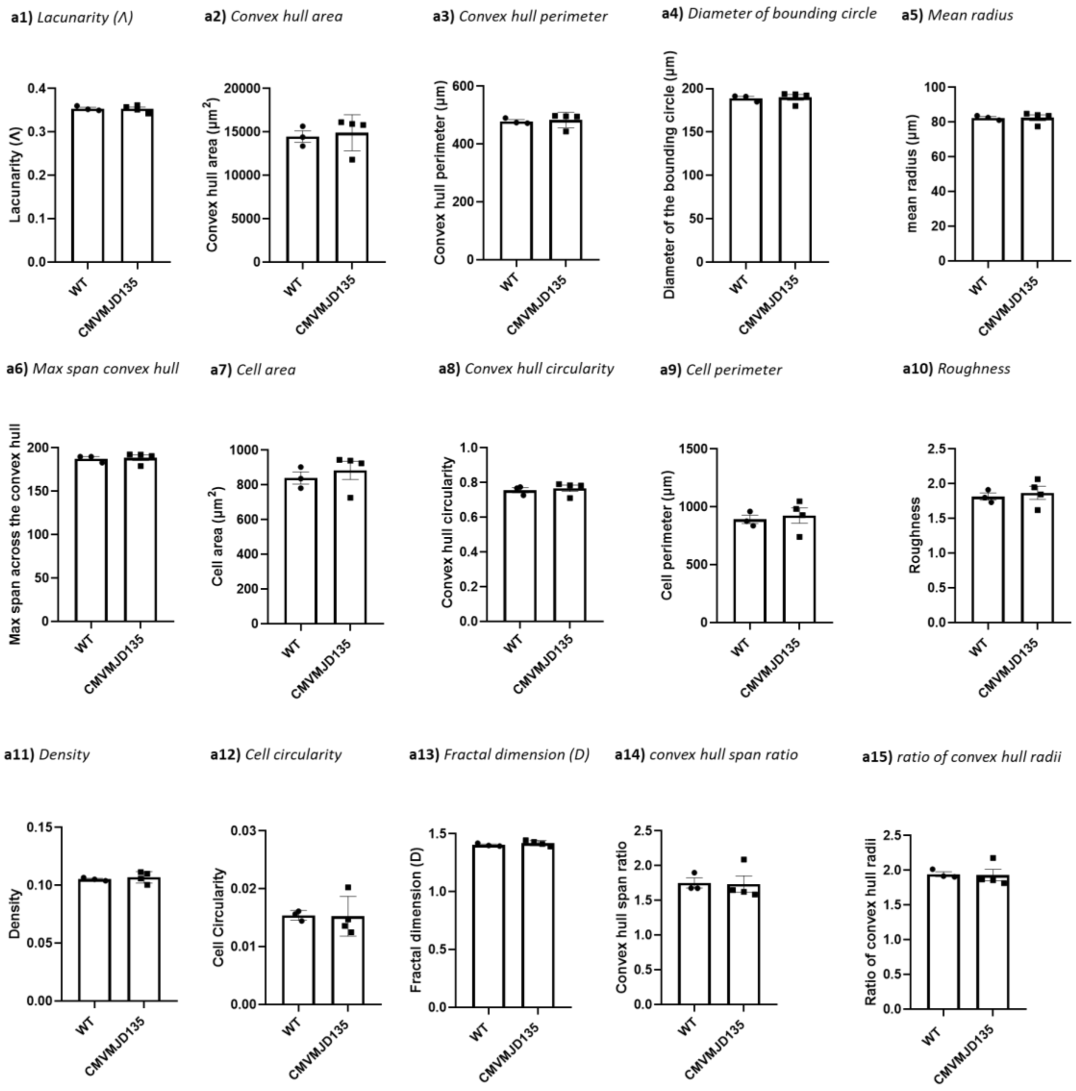

Supplementary Figure S6. The complexity and shape of microglia in the PN of CMVMJD135 mice are similar to those of microglia from WT mice. a) Quantification of the morphometric parameters associated with the heterogeneity of the shape: **a1)** lacunarity (Λ); associated with cell size: **a2)** convex hull area**, a3)** the convex hull perimeter**, a4)** the diameter of bounding circle, **a5)** the mean radius, **a6)** the maximum span across the convex hull, **a7)** the cell area, and **a8)** the convex hull circularity; associated with cell surface **a9)** cell perimeter and **a10)** roughness**;** associated with soma thickness: **a11)** density and **a12)** cell circularity; associated with the complexity of ramifications: **a13)** fractal dimension (D); and associated with the cylindrical shape of cells: **a14)** convex hull span ratio and **a15)** the ratio of convex hull radii. Values for all these parameters were obtained from 152 microglial cells from WT mice (n = 3) and 180 microglial cells from CMVMJD135 mice (n = 4) of the PN. Data are presented as mean+SEM (Student's t-test).

Morphological analysis of microglia from the DCN of CMVMJD135 and WT mice

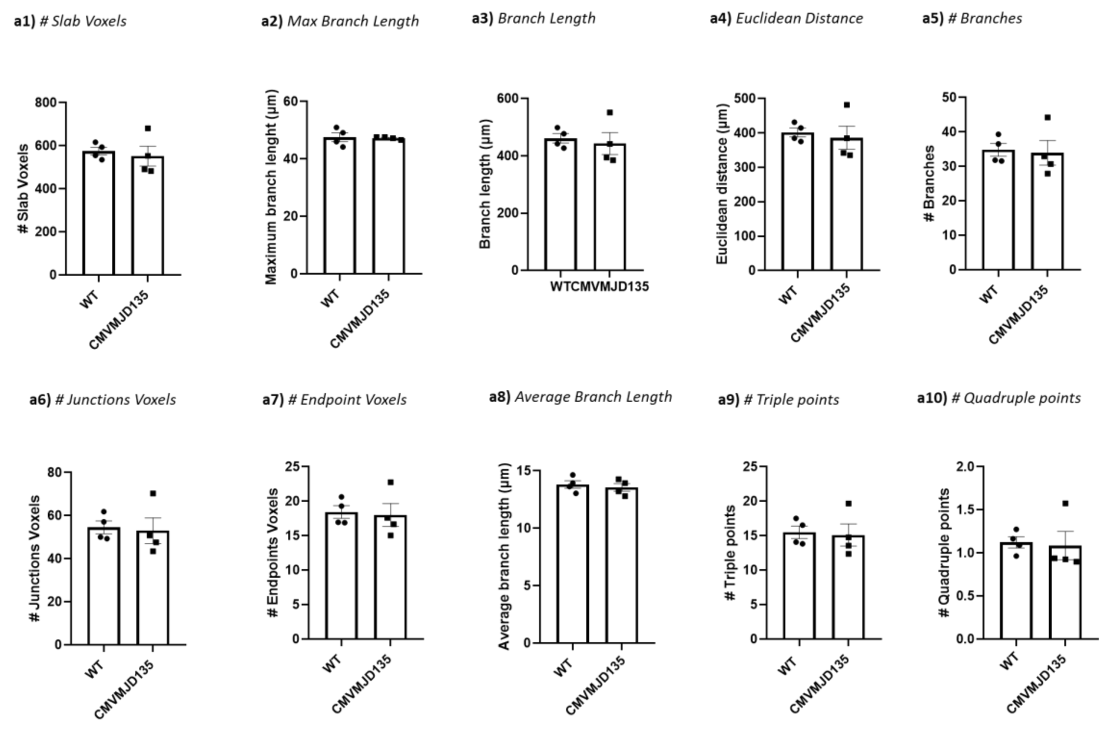

Supplementary Figure S7. Microglia in the deep cerebellar nuclei (DCN) of CMVMJD135 mice showed no differences in features relevant to microglia ramification. a) Quantification of the morphometric parameters associated with microglia ramification including: **a1)** # slab voxels; **a2)** maximum branch length**; a3)** branch length; **a4)** Euclidean distance; **a5)** # branches**; a6)** # junctions' voxels; **a7)** # endpoints voxels; **a8)** average branch length; **a9)** # triple points**;** and **a10)** # quadruple points. Values for all these parameters were obtained from 349 microglial cells from WT mice (n = 4) and 445 microglial cells from CMVMJD135 mice (n = 4) of the DCN. Data are presented as mean+SEM (Student's t-test).

Morphological analysis of microglia from the DCN of CMVMJD135 and WT mice

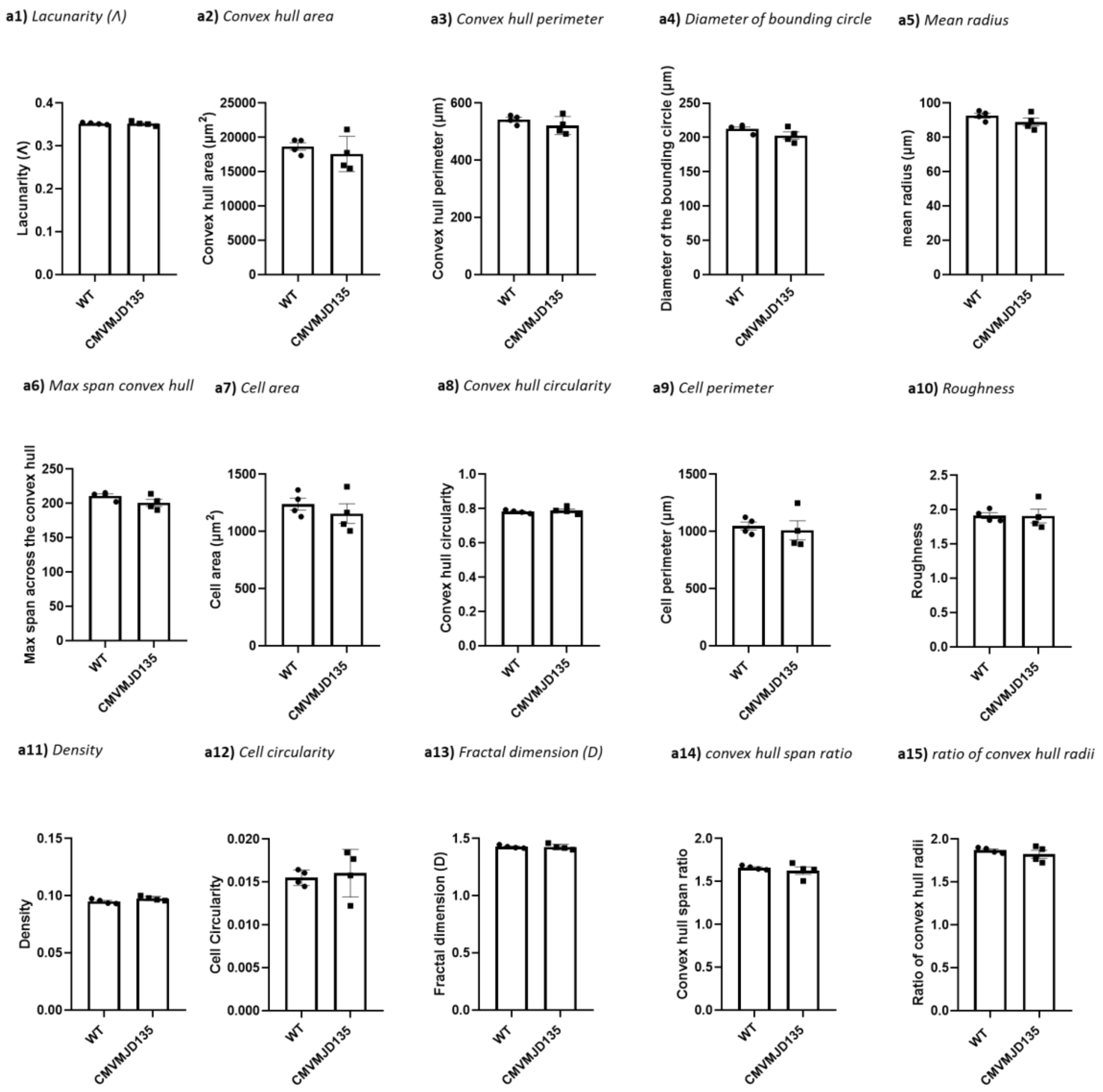

Supplementary Figure S8. Microglia in the DCN of CMVMJD135 mice showed no changes in the complexity and shape. a) Quantification of the morphometric parameters associated with heterogeneity of the shape: **a1)** lacunarity (Λ); associated with cell size: **a2)** convex hull area**, a3)** the convex hull perimeter**, a4)** the diameter of bounding circle, **a5)** the mean radius, **a6)** the maximum span across the convex hull, **a7)** the cell area, and **a8)** the convex hull circularity; associated with cell surface: **a9)** cell perimeter and **a10)** roughness**;** associated with soma thickness: **a11)** density and **a12)** cell circularity; associated with the complexity of ramifications: **a13)** fractal dimension (D); and associated cylindrical shape of the cells: **a14)** convex hull span ratio and **a15)** the ratio of convex hull radii. Values for all these parameters were obtained from 349 microglial cells from WT mice (n = 4) and 445 microglial cells from CMVMJD135 mice (n = 4) of the DCN. Data are presented as mean+SEM (Student's t-test).

<u>**Morphological analysis of microglia from the CSC of CMVMJD135 and WT mice**</u>

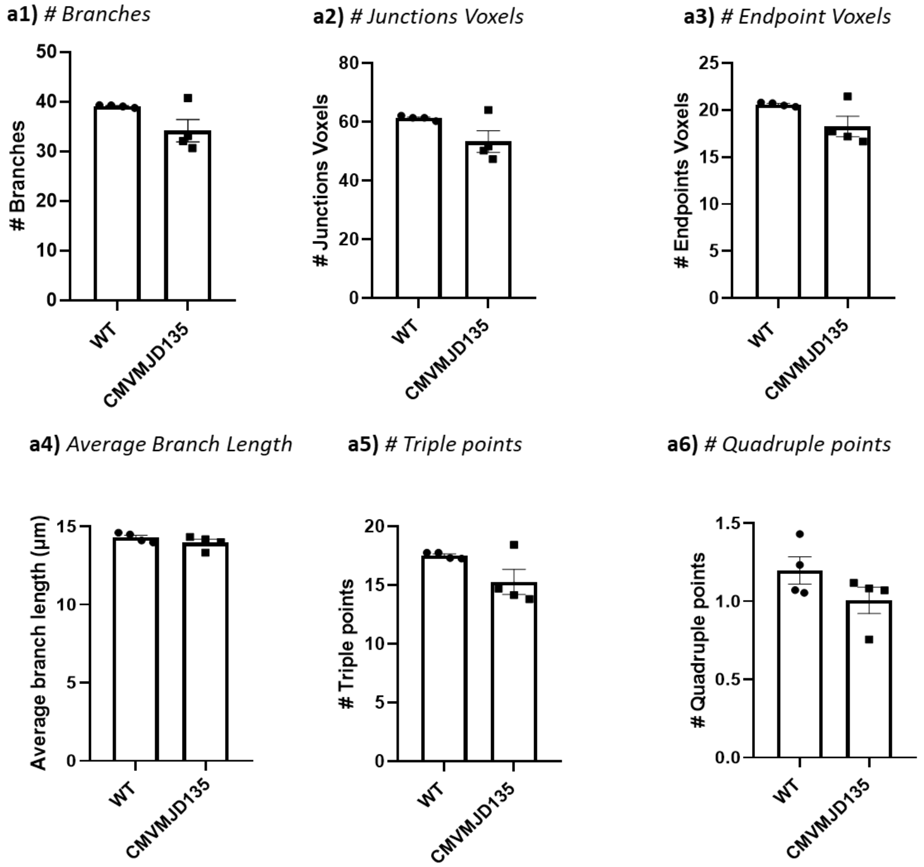

Supplementary Figure S9. Some parameters associated with microglia ramification were similar between CMVMJD135 and WT mice in the cervical spinal cord (CSC). **a)** Quantification of the morphometric parameters associated with microglia ramification including: **a1)** # branches**; a2)** # junctions' voxels; **a3)** # endpoints voxels; **a4)** average branch length; **a5)** # triple points**;** and **a6)** # quadruple points. Values for all these parameters were obtained from 310 microglial cells from WT mice (n = 4) and 389 microglial cells from CMVMJD135 mice (n = 4) of the CSC. Data are presented as mean+SEM (Student's t-test).

Morphological analysis of microglia from the CSC of CMVMJD135 and WT mice

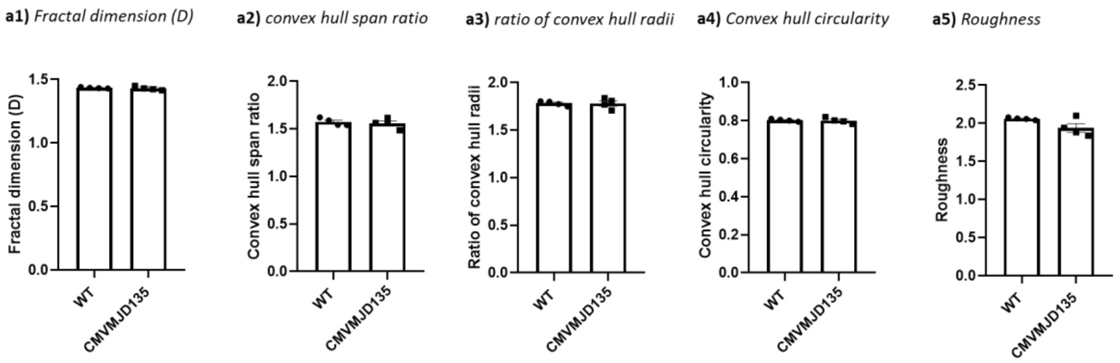

Supplementary Figure S10. No changes were observed in the parameters related to the complexity of ramifications and with the cylindrical shape of the cells between groups in the CSC. a) Quantification of the morphometric parameters associated with the complexity of ramifications: **a1)** fractal dimension (D); associated with cylindrical shape of the cells: **a2)** convex hull span ratio and **a3)** the ratio of convex hull radii; one of the parameters associated with cell's size: **a4)** the convex hull circularity; and one of the parameters associated with cell's surface: **a5)** roughness**.** Values for all these parameters were obtained from 310 microglial cells from WT mice (n = 4) and 389 microglial cells from CMVMJD135 mice (n = 4) of the CSC. Data are presented as mean+SEM (Student's t-test).

Evaluation of microglial enrichment in samples from CMVMJD135 and WT mice

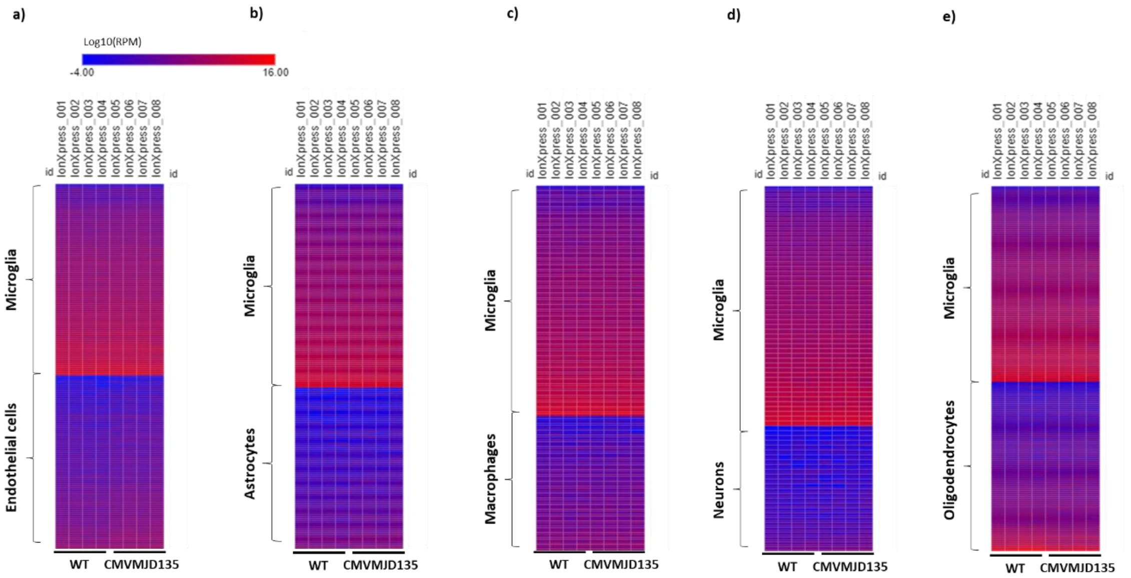

Supplementary Figure S11. Evaluation of microglial enrichment in RNA-sequencing samples. Heatmaps showing high levels of expression for specific markers of microglia when compared with markers of other cell-types. a) Microglia versus endothelial cells; b) microglia versus astrocytes; c) microglia versus macrophages; d) microglia versus neurons; and e) microglia versus oligodendrocytes. Four biological replicates for WT and CMVMJD135 mice were used. An heatmap containing the cell-specific markers was achieved using the Clue Morpheus software.

<u>**RNA-sequencing analysis of microglia from CMVMJD135 and WT mice**</u>

a)

ID count: 83	Fold Chan...	P-val	FDR P-val	Public Gene IDs	Gene Symbol
Ufl1	1,72	1,63E-06	0,0097	NM_026194	Ufl1
Lrrc58	1,49	3,44E-06	0,0137	NM_177093	Lrrc58
Lamc1	2,5	4,34E-06	0,0149	NM_010683	Lamc1
Cdyl	1,95	8,26E-06	0,0247	NM_001123386	Cdyl
Rnf144b	1,54	1,37E-05	0,0364	NM_001170643	Rnf144b
Ncam1	2	1,55E-05	0,0367	NM_001081445	Ncam1
Ddb1	1,47	1,75E-05	0,0367	NM_015735	Ddb1
Cux2	2,36	1,84E-05	0,0367	NM_001312908	Cux2
A2m	1,97	2,29E-05	0,0391	NM_175628	A2m
Epsti1	1,28	3,46E-05	0,0552	NM_029495	Epsti1
Hipk3	1,71	3,88E-05	0,0581	NM_001145824	Hipk3
Ccdc151	2,36	4,16E-05	0,0581	NM_001163787	Ccdc151
Arhgef12	1,79	4,49E-05	0,0581	NM_027144	Arhgef12
Pcdhb21	2,83	4,77E-05	0,0581	NM_053146	Pcdhb21
Mef2c	1,43	5,24E-05	0,0597	NM_001170537	Mef2c
Mira	1,67	5,88E-05	0,0639	NR_045199	Mira
L3mbtl3	1,98	6,73E-05	0,0686	NM_172787	L3mbtl3
Fam188b	1,62	6,88E-05	0,0686	NM_001142781	Fam188b
Gm6548	2,33	7,64E-05	0,0697	NR_003363	Gm6548
Tmem136	2,02	7,78E-05	0,0697	NM_001034863	Tmem136
4930564C03Rik	1,72	7,86E-05	0,0697	NM_029257	4930564C03Rik
Pcnx3	1,82	0,0001	0,0817	NM_144868	Pcnx3
Scd2	1,74	0,0001	0,0817	NM_009128	Scd2
Haghl	1,48	0,0001	0,0817	NM_001271433	Haghl
Zfp568	2,01	0,0001	0,0850	NM_001033355	Zfp568
Ak8	1,98	0,0001	0,0850	NM_001033874	Ak8
Mpg	1,69	0,0001	0,0850	NM_010822	Mpg
Ap3m2	1,61	0,0001	0,0850	NM_001122820	Ap3m2
Sox8	2,07	0,0001	0,0850	NM_011447	Sox8
Rbbp6	1,56	0,0001	0,0850	NM_011247	Rbbp6
Ptpn4	1,78	0,0001	0,0850	NM_019933	Ptpn4
Nav1	1,9	0,0002	0,0850	NM_173437	Nav1
Abcb1a	2,25	0,0002	0,0850	NM_011076	Abcb1a
Igfbp3	5,15	0,0002	0,0850	NM_008343	Igfbp3
Celsr1	2,81	0,0002	0,0850	NM_009886	Celsr1
Dok7	4,17	0,0002	0,0850	NM_172708	Dok7
Zc3h6	2,97	0,0002	0,0850	NM_178404	Zc3h6
Hydin	2,39	0,0002	0,0850	NM_172916	Hydin
Alpl	2,13	0,0002	0,0850	NM_001287172	Alpl
Abca7	1,7	0,0002	0,0850	NM_013850	Abca7
Syt3	1,48	0,0002	0,0850	NM_001114116	Syt3
St8sia4	1,63	0,0002	0,0850	NM_001159745	St8sia4
Gsk3b	1,59	0,0002	0,0850	NM_019827	Gsk3b
Acsl4	1,41	0,0002	0,0850	NM_001033600	Acsl4
Csad	1,27	0,0002	0,0850	NM_144942	Csad
Tanc1	1,43	0,0002	0,0850	NM_001290659	Tanc1
St5	1,5	0,0002	0,0867	NM_001001326	St5
Tmem106b	1,33	0,0002	0,0867	NM_027992	Tmem106b
Prrc2a	1,76	0,0002	0,0906	NM_001199044	Prrc2a
Klhl24	1,39	0,0002	0,0906	NM_029436	Klhl24
Sh2d5	1,56	0,0002	0,0906	NM_001099631	Sh2d5
Rad54l2	1,57	0,0002	0,0906	NM_030730	Rad54l2
Zfp882	1,26	0,0003	0,0930	NM_001166645	Zfp882
Degs2	2,15	0,0003	0,0930	NM_001171002	Degs2
Vps37c	1,3	0,0003	0,0930	NM_181403	Vps37c
Bmpr2	1,99	0,0003	0,0930	NM_007561	Bmpr2
Arhgap20	1,94	0,0003	0,0930	NM_175535	Arhgap20
Xpr1	1,23	0,0003	0,0930	NM_011273	Xpr1
Caskin2	1,85	0,0003	0,0934	NM_080643	Caskin2
Gpld1	1,55	0,0003	0,0935	NM_008156	Gpld1
Ola1	1,27	0,0003	0,0954	NM_025942	Ola1
Foxf2	2,13	0,0003	0,0961	NM_010225	Foxf2
Cpd	1,62	0,0003	0,0961	NM_007754	Cpd
Cpsf7	1,63	0,0003	0,0961	NM_001164272	Cpsf7
1110004F10Rik	1,56	0,0003	0,0961	NM_019772	1110004F10Rik
Kat6a	1,22	0,0003	0,0961	NM_001081149	Kat6a
Mkl2	3,3	0,0003	0,0961	NM_001122667	Mkl2
Map3k19	1,64	0,0004	0,0995	NM_011737	Map3k19
Tyro3	2,17	0,0004	0,0995	NM_001290800	Tyro3
Fmnl2	1,59	0,0004	0,0995	NM_172409	Fmnl2
Atp2b4	1,72	0,0004	0,0995	NM_001167949	Atp2b4
Junb	1,45	0,0004	0,0995	NM_008416	Junb
Atp6v0a1	1,46	0,0004	0,0995	NM_001243049	Atp6v0a1
Atg101	1,41	0,0004	0,0995	NM_026566	Atg101
Frmd4b	1,36	0,0004	0,0995	NM_145148	Frmd4b
Cnot1	1,34	0,0004	0,0995	NM_001205226	Cnot1
Ahnak	1,97	0,0004	0,0995	NM_001039959	Ahnak
Arhgef15	2,48	0,0004	0,0995	NM_177566	Arhgef15
Ntn1	2,78	0,0004	0,0995	NM_008744	Ntn1
Fos	1,47	0,0004	0,0995	NM_010234	Fos
Rnh1	1,21	0,0004	0,0995	NM_001172100	Rnh1
Fbxl12	1,76	0,0004	0,0995	NM_001002846	Fbxl12
Stard13	2,16	0,0004	0,0995	NM_001163493	Stard13

b)

ID count: 18	Fold Chan...	P-val	FDR P-val	Public Gene IDs	Gene Symbol
4921530L21Rik	-1,09	1,98E-08	0,0002	NM_025733	4921530L21Rik
Cpsf1	-1,09	1,98E-08	0,0002	NM_001164173	Cpsf1
Olfr791	-1,09	1,98E-08	0,0002	NM_146930	Olfr791
Zfp62	-1,98	2,04E-06	0,0097	NM_001024846	Zfp62
Bend6	-1,97	2,16E-05	0,0391	NM_001310484	Bend6
Usp11	-1,28	4,85E-05	0,0581	NM_145628	Usp11
Gm2694	-6,97	9,62E-05	0,0817	NR_033430	Gm2694
LOC108169012	-4,19	0,0001	0,0841	XR_001784563	LOC108169012
Akap7	-3,22	0,0001	0,0850	NM_018747	Akap7
Fbxw4	-1,68	0,0002	0,0850	NM_013907	Fbxw4
Nek9	-1,22	0,0002	0,0850	NM_145138	Nek9
Hoxb8	-1,63	0,0002	0,0906	NM_010461	Hoxb8
Phlpp1	-1,67	0,0003	0,0930	NM_133821	Phlpp1
Plin2	-1,41	0,0003	0,0930	NM_007408	Plin2
Pnpla3	-1,63	0,0003	0,0930	NM_054088	Pnpla3
Rbfox1	-2,21	0,0003	0,0930	NM_021477	Rbfox1
Tasp1	-1,72	0,0003	0,0930	NM_001159640	Tasp1
Tbkbp1	-1,34	0,0003	0,0954	NM_198100	Tbkbp1

c)

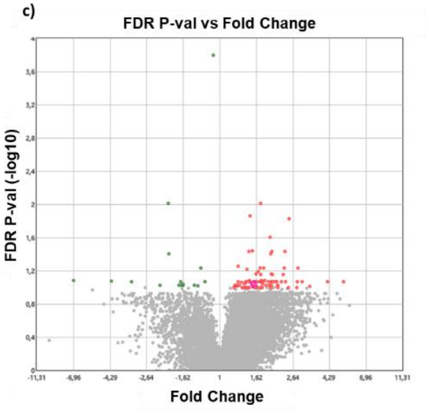

Supplementary Figure S12. Differential gene expression between microglial cells from CMVMJD135 and WT mice. a, b, c) Up-regulated and down-regulated genes were determined using the Transcriptome Analysis Console (TAC) software, between CMVMJD135 and WT mice. **a)** Heatmap of 83 up-regulated genes in microglial cells from CMVMJD135 mice, in ascending order of False Discovery Rate (FDR) value. **b)** Heatmap of 18 down-regulated genes in microglial cells from CMVMJD135 mice, in ascending order of FDR value. **c)** Volcano plot view of CMVMJD135 versus WT genes. Red for up-regulated genes and green for down-regulated genes. |fold change| > 1, $p < 0.05$, and an FDR < 0.1 was considered to determine genes significantly differentially expressed.

Transcriptional changes seen in CMVMJD135 microglia overlap those in Amyotrophic lateral sclerosis (ALS) and Alzheimer disease (AD) mouse models

a)

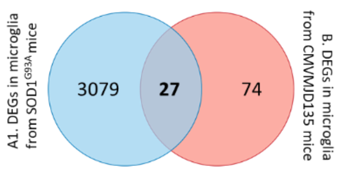

27 Overlapping Genes	A1	B
Lamc1	↑	↑
Hipk3	↑	↑
Lrrc58	↑	↑
Bmpr2	↑	↑
Nav1	↑	↑
St8sia4	↑	↑
Cpd	↑	↑
Fmnl2	↑	↑
Atp6v0a1	↑	↑
Klhl24	↑	↑
Cnot1	↑	↑
Tmem106b	↑	↑
Xpr1	↑	↑
Rnh1	↑	↑
Bend6	↓	↓
Ups11	↓	↓
Tbkbp1	↓	↓
Ncam1	↓	↑
Arhgef15	↓	↑
Abcb1a	↓	↑
Alpl	↓	↑
Foxf2	↓	↑
Caskin2	↓	↑
Fbxl12	↓	↑
Gpld1	↓	↑
Csad	↓	↑
Plin2	↑	↓

p = 0.0003, OR = 2.459
Sobue et al., 2021

b)

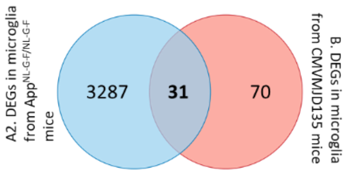

31 Overlapping Genes	A2	B
Gm6548	↑	↑
Rnf144b	↑	↑
Epsti1	↑	↑
St8sia4	↑	↑
Cpd	↑	↑
Fos	↑	↑
Junb	↑	↑
Acsl4	↑	↑
Klhl24	↑	↑
Bend6	↓	↓
Phlpp1	↓	↓
Rbfox1	↓	↓
Cux2	↓	↑
Ncam1	↓	↑
Arhgef12	↓	↑
Mkl2	↓	↑
Arhgef15	↓	↑
Abcb1a	↓	↑
Tyro3	↓	↑
Alpl	↓	↑
Foxf2	↓	↑
Sox8	↓	↑
Ahnak	↓	↑
Caskin2	↓	↑
Scd2	↓	↑
Atp2b4	↓	↑
Sh2d5	↓	↑
Gpld1	↓	↑
Syt3	↓	↑
Plin2	↑	↓
Klhl24	↑	↓

p = <0.0001, OR = 2.768
Sobue et al., 2021

c)

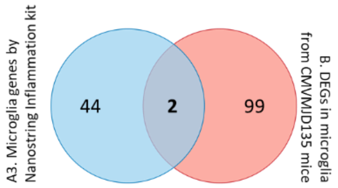

2 Overlapping Genes	A3	B
Mef2c	↑	↑
Fos	↑	↑

p = 0.0162, OR = 10.92
Butovsky et al., 2014

Supplementary Figure S13. Transcriptional changes seen in CMVMJD135 microglia overlap those in Amyotrophic lateral sclerosis (ALS) and Alzheimer disease (AD) mouse models. Venn diagrams and table overview representing the overlapping genes between the 101 CMVMJD135-altered genes found in our RNA-sequencing analysis, with **a)** 3106 differentially expressed genes (DEGs) previously reported in the microglia of a mouse model of ALS, $SOD1^{G93A}$ mouse [51]; with **b)** 3318 DEGs previously reported in the microglia of a mouse model of AD, AppNL-G-F/NL-G-F mouse [51]; and with **c)** 46 microglial genes highly expressed and/or affected in microglia in different neuroinflammatory conditions, as detected by the Nanostring inflammation kit [64]. Red arrows represent the up-regulated genes and green arrows represent the down-regulated genes. Comparisons were conducted by contingency analysis, using the Fisher's exact test and the Baptista-Pike method to calculate the odds-ratio. Significance was set at $p < 0.05$.

2. Supplementary References

[51] Sobue, A.; Komine, O.; Hara, Y.; Endo, F.; Mizoguchi, H.; Watanabe, S.; Murayama, S.; Saito, T.; Saido, T.C.; Sahara, N.; Higuchi, M.; Ogi, T.; Yamanaka, K. Microglial gene signature reveals loss of homeostatic microglia associated with neurodegeneration of Alzheimer´s disease. Acta Neuropathologica Communications 2021, 9. doi:10.1186/s40478-020-01099-x.

[64] Butovsky, O.; Jedrychowski, M.P.; Moore, C.S.; Cialic, R.; Lanser, A.J.; Gabriely, G.; Koeglsperger, T.; Dake, B.; Wu, P.M.; Doykan, C.E.; Fanek, Z.; Liu, L.; Chen, Z.; Rothstein, J.D.; Ransohoff, R.M.; Gygi, S.P.; Antel, J.P.; Weiner, H.L. Identification of a unique TGF-β-dependent molecular and functional signature in microglia. Nature Neuroscience 2014, 17, 131–43. doi:10.1038/nn.3599.
